# Health Insurance Coverage and Postpartum Outcomes in the US: A Systematic Review

**DOI:** 10.1001/jamanetworkopen.2023.16536

**Published:** 2023-06-02

**Authors:** Ian J. Saldanha, Gaelen P. Adam, Ghid Kanaan, Michael L. Zahradnik, Dale W. Steele, Kenneth K. Chen, Alex F. Peahl, Valery A. Danilack-Fekete, Alison M. Stuebe, Ethan M. Balk

**Affiliations:** 1 Center for Clinical Trials and Evidence Synthesis, Department of Epidemiology, Johns Hopkins Bloomberg School of Public Health, Baltimore, Maryland; 2 Center for Evidence Synthesis in Health, Department of Health Services, Policy, and Practice, Brown University School of Public Health, Providence, Rhode Island; 3 Departments of Emergency Medicine and Pediatrics, Brown University Warren Alpert Medical School, Providence, Rhode Island; 4 Department of Medicine, Department of Obstetrics and Gynecology, Brown University Warren Alpert Medical School, Providence, Rhode Island; 5 Department of Obstetrics and Gynecology, University of Michigan, Ann Arbor; 6 Center for Outcomes Research and Evaluation, Department of Internal Medicine, Yale University School of Medicine, New Haven, Connecticut; 7 Department of Epidemiology, Brown University School of Public Health, Providence, Rhode Island; 8 Department of Obstetrics and Gynecology, University of North Carolina School of Medicine, Chapel Hill

## Abstract

**Importance:**

Approximately half of postpartum individuals in the US do not receive any routine postpartum health care. Currently, federal Medicaid coverage for pregnant individuals lapses after the last day of the month in which the 60th postpartum day occurs, which limits longer-term postpartum care.

**Objective:**

To assess whether health insurance coverage extension or improvements in access to health care are associated with postpartum health care utilization and maternal outcomes within 1 year post partum.

**Evidence Review:**

Medline, Embase, CENTRAL, CINAHL, and ClinicalTrials.gov were searched for US-based studies from inception to November 16, 2022. The reference lists of relevant systematic reviews were scanned for potentially eligible studies. Risk of bias was assessed using questions from the Cochrane Risk of Bias tool and the Risk of Bias in Nonrandomized Studies of Interventions tool. Strength of evidence (SoE) was assessed using the Agency for Healthcare Research and Quality Methods Guide.

**Findings:**

A total of 25 973 citations were screened and 28 mostly moderate-risk-of-bias nonrandomized studies were included (3 423 781 participants) that addressed insurance type (4 studies), policy changes that made insurance more comprehensive (13 studies), policy changes that made insurance less comprehensive (2 studies), and Medicaid expansion (9 studies). Findings with moderate SoE suggested that more comprehensive association was likely associated with greater attendance at postpartum visits. Findings with low SoE indicated a possible association between more comprehensive insurance and fewer preventable readmissions and emergency department visits.

**Conclusions and Relevance:**

The findings of this systematic review suggest that evidence evaluating insurance coverage and postpartum visit attendance and unplanned care utilization is, at best, of moderate SoE. Future research should evaluate clinical outcomes associated with more comprehensive insurance coverage.

## Key Points

**Question:**

Are health insurance coverage extension or improvements in access to health care associated with postpartum health care utilization and maternal outcomes within 1 year post partum?

**Findings:**

This systematic review included 28 mostly moderate-risk-of-bias nonrandomized studies. An association between more comprehensive insurance and greater attendance at postpartum visits was observed in some studies based on a moderate strength of evidence; for other types of postpartum health care outcomes, the strength of evidence for association with insurance coverage improvement was low.

**Meaning:**

These findings suggest that evidence identified is, at best, of moderate strength; future research should evaluate the impact of more comprehensive or extended health insurance on health outcomes in the postpartum period and beyond.

## Teaser

This systematic review evaluates the association between health insurance coverage extensions or improvements and postpartum outcomes in the US.

## Introduction

Maternal morbidity and mortality have increased considerably in the US.^1^ In 2020, the maternal mortality ratio was 23.8 per 100 000 live births (highest among industrialized countries), with wide racial and ethnic gaps (eg, non-Hispanic Black: 55.3 deaths per 100 000 live births, non-Hispanic White: 19.1, and Hispanic: 18.2).^2^ More than 80% of pregnancy-related deaths are preventable,^3,4^ with various related contributors, such as racism,^5^ system factors (eg, lack of coordination among practitioners), practitioner factors (eg, ineffective treatment), and patient/family factors (eg, poor knowledge about warning signs).

According to recent estimates, approximately 65% of pregnancy-related deaths in the US occur in the first year after giving birth.^4^ Among these, 12% occur within 6 days after delivery, 23% occur 7 to 42 days, and 30% occur 43 days to 1 year.^4^ The previously mentioned factors, including system-level factors, are associated with postpartum deaths. About half of postpartum individuals in the US do not receive routine postpartum health care.^6,7,8,9^ Even for those with access, care may be limited by existing payment models that afford variable coverage for key services. The increasingly common global reimbursement models, in which practitioners receive bundled payments for postpartum care regardless of the number of postpartum visits,^10^ may also disincentivize adequate postpartum care.^11,12^

Currently, federal Medicaid coverage for pregnant individuals lapses after the last day of the month in which the 60th postpartum day occurs,^13^ which limits longer-term postpartum care. The American Rescue Plan Act of 2021 allowed states to request a waiver to extend postpartum Medicaid coverage up to 1 year after delivery.^14^ As of February 23, 2023, 28 states and the District of Columbia have implemented the approved extensions, 7 states are planning extensions, 3 states are seeking federal approvals through waivers, and 2 states have proposed limited coverage extensions.^15^ Extended coverage for approved states began on April 1, 2022, and is intended to run for 5 years.^14^

We conducted a systematic review for the Agency for Healthcare Research and Quality (AHRQ) and the Patient-Centered Outcomes Research Institute to support the American College of Obstetricians and Gynecologists (ACOG) in the development of new guidance on postpartum care of individuals within 1 year after giving birth. The full report addressed questions pertaining to alternative strategies for postpartum health care delivery and extension of postpartum health insurance coverage. Here, we address the second question: are extension of health insurance coverage or improvements in access to health care associated with postpartum health care utilization and maternal outcomes within 1 year post partum? Our outcomes of interest included health care utilization outcomes (eg, attendance at postpartum visits), clinical outcomes (eg, maternal mortality), and harms (eg, worsening health inequities) (Box). We evaluated whether outcomes vary by several patient-level factors (eg, age, race and ethnicity, socioeconomic status) and setting factors (eg, geographic location, different levels of neighborhood vulnerability).

Box. **List of Outcomes and Potential Variables of Interest**

**Outcomes**

Healthcare utilization outcomes
  Attendance at postpartum visits^a^[Boxed-text zoi230502b1]
  Unplanned care utilization (eg, unplanned readmissions, emergency department visits)^a^[Boxed-text zoi230502b1]
  Adherence to condition-specific screening/testing (eg, blood pressure monitoring, glucose tolerance testing) or treatment^a^[Boxed-text zoi230502b1]
  Transition to primary care practitioner for long-term care^a^[Boxed-text zoi230502b1]
Clinical outcomes (as appropriate, outcomes include incidence, prevalence/continuation, severity, and resolution)
  Maternal mortality^a^[Boxed-text zoi230502b1]
  Symptoms or diagnosis of mental health conditions (eg, anxiety, depression, substance use)^a^[Boxed-text zoi230502b1]
  Patient-reported outcomes
    Quality of life (using validated measures)^a^[Boxed-text zoi230502b1]
    Perceived stress^a^[Boxed-text zoi230502b1]
    Pain
    Sleep quality
    Fatigue
    Sexual well-being and satisfaction
    Awareness of risk factors for long-term ill health
  Physical health/medical outcomes
    Postpartum onset of preeclampsia or hypertension
    Infections (eg, mastitis, wound infections)
    Severe maternal morbidity
      Cardiovascular disorders (eg, cardiomyopathy)
      Cerebrovascular disorders (eg, stroke)
      Bleeding
      Venous thromboembolism
      Other
  Interpregnancy interval
  Unplanned pregnancies
  Contraceptive initiation and continuation
  Breastfeeding intention, initiation, duration, and exclusivity
  Reduction in health inequities (eg, by race, ethnicity, geography, disability status)
Harms
  Health inequities^a^[Boxed-text zoi230502b1]
  Reported discrimination^a^[Boxed-text zoi230502b1]
  Over-utilization of health care
  Patient burden regarding postpartum care

**Potential Associated Variables**

Patient-level factors
  Age
  Race and ethnicity
  Gender identity
  Sexual identity
  Physical disability status
  Education level
  Socioeconomic status
  Immigration status
  Refugee status
  Barriers to transportation to health care facility
  Paid family leave policies (eg, presence vs absence, different durations of leave)
  Access to internet (for virtual care/telehealth questions)
  Substance use/substance use disorder
  Type of insurance coverage (insured vs uninsured, private vs public [eg, Medicaid], insurance coverage of postpartum care, Medicaid insurance coverage extension or expansion)
  Presence vs absence of disorders of pregnancy (eg, hypertensive, cardiovascular, gestational diabetes mellitus) or peripartum complications that increase risk of postpartum complications
  Preterm vs term delivery
  Live birth vs stillbirth/spontaneous abortion/induced abortion
  Number of infants (singleton vs twins/triplets, and so forth)
  Presence vs absence of a supportive partner
  Infant health (eg, neonatal intensive care unit [NICU] admission, congenital anomalies)
Setting factors
  Country (US vs Canada)
  Geographic location (urban vs suburban vs rural)
  Different levels of neighborhood vulnerability (eg, social vulnerability index)
  Volume of facility/hospital (high vs low)
  Type of facility/hospital (private vs public, community vs tertiary, academic vs nonacademic)
  Racial/ethnic concordance between practitioner and patient
  Language concordance between practitioner and patient

^a^
Outcomes prioritized for assessment of strength of evidence and for making conclusions.

## Methods

We used standard systematic review methodology as outlined in AHRQ’s Methods Guide.^16^ We refined the research questions, eligibility criteria (including outcomes of interest), and planned methods after discussions with diverse groups of clinical and methodological experts and patient representatives. We prospectively registered the systematic review protocol through PROSPERO. This systematic review is reported in accordance with the Preferred Reporting Items for Systematic Reviews and Meta-analyses (PRISMA) reporting guideline 2020 statement. Institutional review board and informed consent were not relevant for this systematic review because it relied on published information.

### Search Strategy

We searched for published studies in Medline (via PubMed), Embase, the Cochrane Central Register of Controlled Trials, and Cumulative Index to the Nursing and Allied Health Literature, and for unpublished studies with reported results in ClinicalTrials.gov. The searches included terms related to post partum, insurance coverage, and health care strategies (to address both research questions in the report) (eAppendix 1 in Supplement 1). All searches are current as of November 16, 2022. We also scanned the reference lists of relevant systematic reviews for potentially eligible studies.

### Study Selection

Eight investigators (I.J.S., G.P.A., G.K., M.L.Z., D.W.S., A.F.P., V.A.D.-F., and E.M.B.) independently screened each title and abstract using Abstrackr.^17^ We rescreened (in duplicate) all accepted citations in full text. At both stages, we resolved discrepancies through full-team discussion or consultation with a third investigator (from among I.J.S., G.P.A., G.K., M.L.Z., D.W.S., A.F.P., V.A.D.-F., and E.M.B.).

We included studies of individuals (of any age) in the postpartum period (within 1 year after giving birth, which we defined as a live birth, intrauterine fetal death/stillbirth, or induced abortion that occurred at 20 or more weeks of gestation) in the US. Postpartum individuals could be healthy (general population) or at increased risk of postpartum complications due to preexisting conditions, pregnancy-related conditions, or newly diagnosed conditions post partum. Studies could address general postpartum care or specific aspects of postpartum care, such as breastfeeding. Herein, we focus on outcomes prioritized by stakeholder panels (Box). Additional (nonprioritized) outcomes (eg, breastfeeding, contraception) are reported in the full report. We considered as eligible randomized controlled trials with at least 10 participants per group and prospective or retrospective nonrandomized comparative studies with adequate statistical adjustment analyses and at least 30 participants per group.

### Risk of Bias Assessment and Data Extraction

One investigator (from among I.J.S., G.K., and M.L.Z.) assessed risk of bias and extracted data for each study into the Systematic Review Data Repository Plus.^18^ A second investigator (from among I.J.S., G.K., and M.L.Z.) verified all extractions. We used questions from the Cochrane Risk of Bias tool^19^ and the Risk of Bias in Nonrandomized Studies of Interventions (ROBINS-I) tool.^20^

### Syntheses

For dichotomous outcomes, we preferentially evaluated risk ratios (RRs). For continuous outcomes, we evaluated net mean differences (NMDs) (ie, difference-in-differences) for outcomes measured at both baseline and postintervention, or mean differences (MDs) for outcomes measured only postintervention. For nonrandomized studies, we considered only reported adjusted analyses (aRR, aNMD, or aMD). We planned random-effects model meta-analyses where there were at least 3 studies reporting results from similar analyses, but the evidence did not allow for this.

### Strength of Evidence Assessment

We assessed strength of evidence (SoE) as per the AHRQ Methods Guide, considering risk of bias, consistency, precision, directness, and sparsity of the evidence.^21^ For each prioritized outcome, we assigned an SoE rating of high, moderate, low, or insufficient. High, moderate, and low grades indicate the degree of confidence we have that the estimate lies close to the true effect; an insufficient rating indicates the strength of the evidence does not warrant an estimation of the true effect.^21^ In accordance with AHRQ guidance,^22,23^ we use qualifying language regarding SoE when communicating conclusions: “probably” for moderate SoE and “may” for low SoE.

## Results

For the full report, our electronic searches yielded 25 973 citations (Figure). We screened 589 full-text articles, of which 28 studies, reported in 29 articles,^24,25,26,27,28,29,30,31,32,33,34,35,36,37,38,39,40,41,42,43,44,45,46,47,48,49,50,51,52^ were eligible for the current systematic review.

**Figure.**
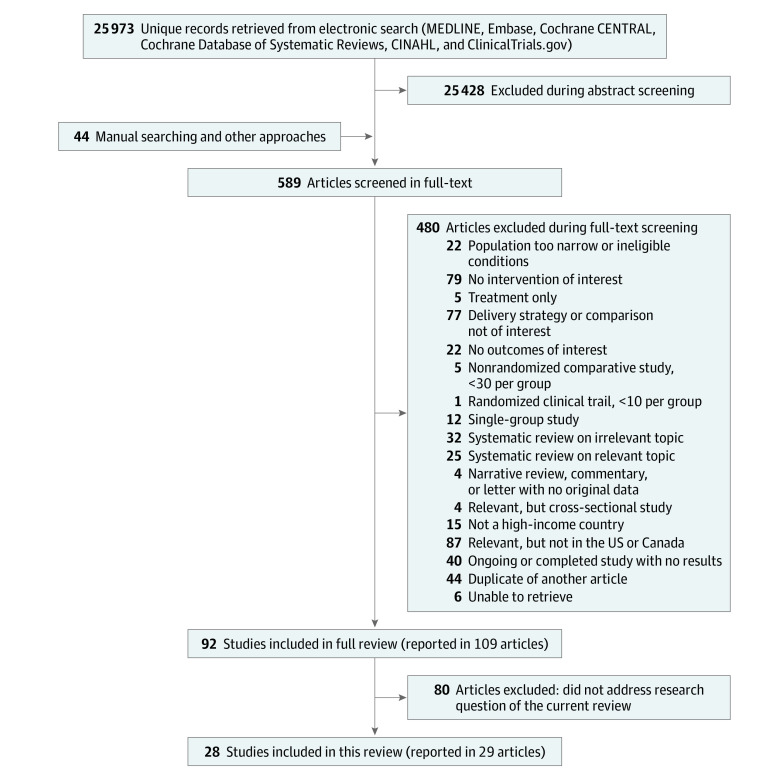
Identification of Studies in This Systematic Review

### Characteristics of Included Evidence

All 28 included studies were nonrandomized comparative studies (published between 2008 and 2022) with adequate statistical adjustment analyses, comprising a total of 3 423 781 postpartum individuals (range 1184 to 1 454 699) (eAppendix 2 in Supplement 1). The studies were conducted in single states (or in the District of Columbia) (16 studies^24,25,27,29,31,32,33,35,38,39,40,41,42,43,44,51,52^), 2 states (4 studies^34,37,48,49^), or 5 or more states (8 studies^26,28,30,36,45,46,47,50^). Fifteen studies focused on general postpartum care and 13 studies focused specifically on contraceptive care.

Study participants were racially diverse, but studies were heterogeneous; between 4% and 83% were White and between 2% and 54% were Black across studies (eAppendix 3 in Supplement 1). Only 1 study reported on employment status; all 2509 participants were employed.^25^ No study reported on participant gender or sexual identity status or on substance use disorders. Where reported, 59% to 74% of births were vaginal and 8% to 22% of births were preterm. Six studies explicitly reported that they excluded pregnancies resulting in stillbirths, spontaneous or induced abortions, or neonatal deaths.

### Comparisons Addressed in Included Evidence

The studies addressed various comparisons (Table 1). For each study, we classified individual comparator groups as more vs less comprehensive insurance coverage. Four studies compared outcomes associated with different types of health insurance. Of these, 2 studies compared private/commercial insurance with Medicaid insurance in Ohio^35^ and North Carolina,^31^ 1 compared continuous Medicaid eligibility with pregnancy-only Medicaid eligibility in Wisconsin,^51^ and 1 compared an insurance plan that fully covered antepartum and postpartum care with a plan that included an annual deductible with out-of-pocket maximums in Massachusetts.^25^ Thirteen studies evaluated the impact of policy changes that made insurance coverage more comprehensive. These included 9 studies of Medicaid expansion in various states^24,33,36,37,39,40,41,42,44,52^; 1 study that evaluated the impact of a law requiring hospitals to provide the option of long-acting reversible contraception (LARC) placement after delivery in Ohio^32^; 1 that evaluated the impact of unbundling (ie, separate reimbursement for immediate postpartum LARC) in Wisconsin^29^; 1 that evaluated the transition from a pilot (Medicaid 1115) expansion of eligibility to individuals otherwise ineligible for Medicaid coverage to the State Plan Amendment, which provides contraceptive care for all, in various states^26^; and 1 study in Texas that evaluated the impact of the Families First Coronavirus Response Act, a federal law that required states to provide continuous coverage to Medicaid enrollees during the COVID-19 pandemic.^43^ In contrast, 2 studies evaluated the impact of policy changes that made insurance coverage less comprehensive: 1 study in North Carolina^38^ that evaluated a policy reducing reimbursement rates for maternity care coordination by 19% and 1 study in Oregon that evaluated a policy requiring undocumented immigrants and legal immigrants within 5 years of immigration with Emergency Medicaid who wanted sterilization following vaginal delivery to pay for it.^27^ Finally, 9 studies compared outcomes in various insurance expansion and nonexpansion (or contraction) states.^28,30,34,45,46,47,48,49,50^

**Table 1.** Summary of Comparisons in 28 Included Studies

Type of comparison (No. studies)	Study, year, PMID	State(s)	Focus of study	More comprehensive insurance	Less comprehensive insurance
Different types of health insurance (4 studies)	Arora, 2018, 29490290	OH	Contraceptive care	Private insurance	Medicaid insurance
	DeSisto, 2020, 32335806	WI	General PP care	Continuous Medicaid eligibility	Pregnancy-only Medicaid eligibility
	Kozhimannil, 2011, 21485419	MA	General PP care	Full coverage of AP and PP care, no cost sharing beyond office visit and hospitalization copayments. Out-patient visit copayments $5-$25 (median $15). Hospitalization copayments $0-$1000 (median $250).	Annual deductible $500-$2000 for individuals and $1000-$4000 for families. Out-of-pocket maximum $2000-$4000 for individuals and $4000-$8000 for families.
	Taylor, 2020, 31397625	NC	General PP care	Commercial insurance	1) Medicaid insurance, 2) No insurance
After vs before a policy change, where the policy change made insurance more comprehensive (13 studies)	Brant, 2021, 34619694	OH	Contraceptive care	Law that required hospitals to offer LARC placement after delivery (2017-2019)	No law that required hospitals to offer LARC placement after delivery (2015-2017)
	Dunlop, 2020, 32958368	OH	Contraceptive care	After Medicaid expansion (2014-2015)	Before Medicaid expansion (2011-2013)
	Koch, 2022, 35588793	MO	Contraceptive care	After Medicaid policy change for separate LARC reimbursement	Before Medicaid policy change for separate LARC reimbursement
	Kramer, 2021, 33849768	WI	Contraceptive care	After unbundling (separate or additional reimbursement for immediate PP LARC)	Before unbundling (no separate or additional reimbursement for immediate PP LARC)
	Liberty, 2020, 31846612	SC	Contraceptive care	After Medicaid policy covering immediate PP LARC (2013-2017)	Before Medicaid policy covering immediate PP LARC (2013-2017)
	Okoroh, 2018, 29530670	IA, LA	Contraceptive care	After Medicaid expansion (2014-2015)	Before Medicaid expansion (2013-2014)
	Redd, 2019, 30484739	MD, MN, MO, NY, OK, OR, PA, WA, WI	Contraceptive care	Transition from the Medicaid 1115 waiver, which allowed states to expand eligibility to some individuals otherwise ineligible for Medicaid coverage, to the State Plan Amendment, which provides contraceptive care to all	Maintenance of the Medicaid 1115 waiver, which allowed states to expand eligibility to some individuals otherwise ineligible for Medicaid coverage
	Schuster, 2022, 34670222	MO, NE, OK, UT, WY	General PP care	After Medicaid expansion (2014-2015)	Before Medicaid expansion (2012-2013)
	Smith, 2021, 34109490	GA	Contraceptive care	After Medicaid policy covering inpatient LARC (2016-2017)	Before Medicaid policy covering inpatient LARC (2015)
	Steenland, 2021a, 33523747	SC	Contraceptive care	After Medicaid policy of payment for immediate PP LARC (2012-2014)	Before Medicaid policy of payment for immediate PP LARC (2011-Jan 2012)
	Steenland, 2021b, 35977301	AR	General PP care	After Medicaid expansion (2014-2015)	Before Medicaid expansion (2013)
	Symum, 2022, 35628011	FL	General PP care	After Statewide Mandatory Medicaid Managed Care (2014-2017)	Before Statewide Mandatory Medicaid Managed Care (2010-2014)
	Wang, 2022, 35592081	TX	General PP care	After Families First Coronavirus Response Act (2020)	Before Families First Coronavirus Response Act (2019)
After vs before a policy change, where the policy change made insurance less comprehensive (2 studies)	Cilenti, 2015, 25627330	NC	General PP care	Before change in Medicaid policy reducing reimbursement rates for maternity care coordination by 19%	After change in Medicaid policy reducing reimbursement rates for maternity care coordination by 19%
	Rodriguez. 2008, 18692614	OR	Contraceptive care	Before policy requiring undocumented immigrants and legal immigrants within 5 y of immigration with Emergency Medicaid to pay for sterilization following vaginal delivery	After policy requiring undocumented immigrants and legal immigrants within 5 y of immigration with Emergency Medicaid to pay for sterilization following vaginal delivery
Insurance expansion vs nonexpansion or contraction states (9 studies)	Austin, 2022, 34974107	20 states	General PP care	Medicaid expansion states	Medicaid nonexpansion states
	Caudillo, 2022, 35488950	16 states	Contraceptive care	Delaware (After Delaware Contraceptive Access Now (DelCAN) initiative)	15 other states (no Delaware Contraceptive Access Now (DelCAN) initiative)
	Eliason, 2021, 34870677	15 states	General PP care	Medicaid expansion states	Medicaid nonexpansion states
	Eliason, 2022, 35259409	11 states	General PP care	Medicaid expansion states	Medicaid nonexpansion states
	Gordon, 2020, 31905073	CO, UT	General PP care	Colorado (after Medicaid expansion)	Utah (no Medicaid expansion)
	Margerison, 2021, 34606358	18 states	General PP care	Medicaid expansion states	Medicaid nonexpansion states
	Myerson, 2020, 33136489	13 states	Contraceptive care	Medicaid expansion states	Medicaid nonexpansion states
	Pace, 2022, 34908011	MA, ME	General PP care	Massachusetts (after Medicaid expansion)	Maine (after Medicaid contraction)
	Rodriguez, 2021, 34910148	OR, SC	General PP care	Oregon (after Medicaid expansion)	South Carolina (no Medicaid expansion)

Abbreviations: AP, antepartum; LARC, long-acting reversible contraception; PMID, PubMed identifier; PP, postpartum.

### Risk of Bias

Nine of the 28 studies had overall high risk of bias due to moderate or serious risk of confounding and the lack of blinding of participants, study staff, and outcome assessors (eAppendix 4 and 5 in Supplement 1). We rated the remaining 19 studies at moderate risk of bias due to the lack of blinding of participants, study staff, and outcome assessors.

### Healthcare Utilization Outcomes

#### Attendance at Postpartum Visits

Eleven studies reported data on attendance at postpartum visits (Table 2 and eAppendix 6 and 7 in Supplement 1).^25,31,33,34,38,39,41,43,48,50,51,52^ Eight studies reported that more comprehensive health insurance was associated with greater attendance. For this and all outcomes, we could not conduct meta-analyses because of the heterogeneity in reported comparisons of insurance (eg, comparisons among insurance types, comparisons of Medicaid expansion vs no expansion) and inconsistent definitions of outcomes (eg, mean number of visits, categorical data on visit attendance).

**Table 2.** Conclusions and Strength of Evidence

Outcome category	Outcome	No. studies (participants)	Risk of bias	Consistency	Precision	Directness	Other	SoE	Conclusions (reason if none)
Healthcare utilization	Attendance at postpartum visits	11 (580 852)	Moderate	Consistent	Precise	Direct	NA	Moderate	More comprehensive insurance associated with greater attendance at postpartum visits
	Unplanned care utilization	1 (1 454 699)	Moderate	NA	Precise	Direct	NA	Low	More comprehensive insurance associated with fewer preventable readmissions and ER visits
	Adherence to screening, testing, or treatment	0	NA	NA	NA	NA	NA	NA	None (no evidence)
	Transition to primary care practitioner for long-term care	0	NA	NA	NA	NA	NA	NA	None (no evidence)
Clinical	Maternal mortality	0	NA	NA	NA	NA	NA	NA	None (no evidence)
	Mental health	3 (149 165)	Moderate	Inconsistent	Precise	Direct	None	Insufficient	None (inconsistent results)
	Quality of life	0	NA	NA	NA	NA	NA	NA	None (no evidence)
	Perceived stress	0	NA	NA	NA	NA	NA	NA	None (no evidence)
Harms	Health inequities	0	NA	NA	NA	NA	NA	NA	None (no evidence)
	Reported discrimination	0	NA	NA	NA	NA	NA	NA	None (no evidence)

Abbreviations: ER, emergency department; NA, not applicable; SoE, strength of evidence.

Three studies reported data on mean number of postpartum visits (eAppendix 6 in Supplement 1). Cilenti et al^38^ reported a higher number of visits per patient by 3 months comparing before vs after a North Carolina Medicaid policy that reduced reimbursement for maternity care coordination by 19% (aMD, 1.6 visits; *P* < .001; no 95% CI reported). Gordon et al^34^ reported that, although the mean numbers of outpatient visits per patient by 1 month were comparable between Colorado and Utah, Colorado (a Medicaid expansion state) had more outpatient visits per patient than Utah (a nonexpansion state) by 3 months (aNMD, 0.10 visits; *P* < .001; no 95% CI reported) and 6 months (aNMD, 0.52; *P* < .01; no 95% CI reported). By 6 months, the mean number of visits was also greater among the subgroup of participants with severe maternal morbidity, such as hemorrhage, acute myocardial infarction, and sepsis (aNMD, 1.25; *P* < .01; no 95% CI reported).^34^ Steenland et al^39,40^ reported that Arkansas’ Medicaid expansion was associated with greater numbers of outpatient visits per patient by 2 months (aMD, 0.2 visits; 95% CI, 0.1 to 0.3) and by 6 months post partum (aMD, 0.9; 95% CI, 0.7 to 1.1).

Eight studies reported categorical data on attendance at postpartum visits (eAppendix 7 in Supplement 1). DeSisto et al^51^ reported that in Wisconsin, compared with participants with pregnancy-only Medicaid coverage, participants with continuous Medicaid eligibility had greater likelihood of the composite outcome of postpartum visit attendance, cervical cytology, intrauterine device (IUD) insertion, or a bundled service (aRD, 6.27%; 95% CI, 5.72 to 6.82) and the composite outcome of postpartum visit attendance, cervical cytology, or IUD insertion (aRD, 12.0%; 95% CI, 11.2 to 12.7). Similarly, Dunlop et al^33^ reported that among income-eligible participants (but not among participants with pregnancy-only Medicaid coverage), Ohio’s Medicaid expansion was associated with greater attendance by 6 months (37.1% after Medicaid expansion vs 31.5% before Medicaid expansion; odds ratio [OR] adjusted marginal effect, 5.09; *P* < .01; no 95% CI reported). Rodriguez et al^48^ reported that Medicaid expansion in Oregon was associated with greater attendance at postpartum visits when compared with South Carolina (a nonexpansion state) (adjusted net prevalence difference 47.9%; 95% CI, 41.3% to 54.6%). Taylor et al^31^ reported that in North Carolina, compared with patients with commercial insurance, attendance at the 6-week visit was lower among patients with Medicaid insurance (adjusted OR [aOR], 0.65; 95% CI, 0.58 to 0.74) and even lower for patients with no insurance (aOR, 0.42; 95% CI, 0.34 to 0.51).

On the other hand, Eliason et al^50^ evaluated 15 states and reported that states with and without Medicaid expansion had comparable attendance at postpartum visits (adjusted net prevalence difference 0.3%; 95%, CI −3.1% to 3.9%). Similarly, Kozhimannil et al,^25^ 2011 evaluated commercial insurance in Massachusetts and reported that participants with an annual deductible with out-of-pocket maximums had comparable attendance at visits between 21 and 56 days post partum as participants who had lower copayments after a policy change (aOR, 0.74; 95% CI, 0.42 to 1.32). Liberty et al^52^ evaluated South Carolina’s Medicaid policy covering immediate postpartum LARC, and Wang et al^43^ evaluated Texas’s Families First Coronavirus Response Act, but neither study reported an adjusted effect size.

#### Unplanned Healthcare Utilization

One study (Symum et al^42^) reported on unplanned health care utilization (eAppendix 7 in Supplement 1). Florida’s Mandatory Medicaid Managed Care policy was associated with a lower rate of preventable readmissions (incidence rate ratio [IRR], 0.86; 95% CI, 0.80 to 0.93) and emergency department visits (IRR, 0.87; 95% CI, 0.82 to 0.93) by 1.5 months post partum.^42^

### Clinical Outcomes

#### Mental Health Outcomes

Three studies reported inconsistent results regarding depression symptoms (“always” or “often” feeling down/depressed/hopeless or had little interest/pleasure in doing things since delivery) (eAppendix 8 in Supplement 1).^28,36,47^ Austin et al,^47^ which evaluated 20 states, and Margerison et al,^28^ which evaluated 18 states, reported that there was no difference in the prevalence of reported depression symptoms in Medicaid expansion and nonexpansion states. However, Schuster et al^36^ reported that Medicaid expansion was associated with a reduction in depression symptoms in 5 states (adjusted prevalence difference, −3.5%; *P* = .04; the prevalence estimates before and after Medicaid expansion were not reported). No study reported on condition-specific screening/testing/treatment or transition to primary care practitioner for long-term care, maternal mortality, quality of life, perceived stress, or harms (health inequities or reported discrimination).

## Discussion

The evidence identified in this systematic review of studies in the US (Table 2) suggests that more comprehensive health insurance coverage is probably associated with greater attendance at postpartum visits (moderate SoE) and may be associated with fewer preventable readmissions and emergency department visits (low SoE). We did not find evidence addressing the other prioritized health care utilization outcomes. There is insufficient evidence on whether more comprehensive insurance is associated with improved symptoms or diagnoses of mental health conditions. We did not find evidence addressing the other prioritized clinical outcomes or harms. Although more comprehensive insurance coverage is probably associated with greater attendance at postpartum visits, the impact of this association on maternal (or child) health outcomes is unclear.

### Implications for Clinical Practice

We found that more comprehensive insurance coverage is probably associated with greater attendance at postpartum visits. In other words, uninsured and underinsured postpartum individuals are less likely to attend their scheduled postpartum visits. Although the evidence was not examined in this systematic review (our focus was on outcomes in the first postpartum year), uninsured and underinsured postpartum individuals are likely more susceptible to poorer outcomes in the long run.

### Implications for Research

Research is needed to evaluate the associations between comprehensiveness of health insurance and outcomes beyond postpartum visit attendance. The ongoing increase in the number of states that are extending postpartum care up to 1 year after delivery^15^ presents an excellent natural experiment and opportunity to examine how these policy changes may (or may not) impact postpartum health outcomes, both within and across states. For example, we are aware of another study by Steenland et al^53^ published after our last search; its results are consistent with our conclusions regarding the associations between Medicaid expansion (ie, more comprehensive insurance coverage) and greater postpartum visit attendance and fewer hospitalizations.^53^ The ongoing research also offers the opportunity to evaluate whether these policy changes may reduce the stark racial and other disparities in postpartum outcomes in the US.

Most studies included in this systematic review enrolled predominantly healthy individuals. Researchers should also design studies that, either entirely or in part, enroll individuals at high risk of postpartum complications due to chronic conditions (eg, preexisting diabetes), pregnancy-related conditions (eg, gestational hypertension) or incident or newly diagnosed conditions (eg, postpartum preeclampsia). There is a particular need to study how marginalized and most at-risk individuals may be impacted by changes to health insurance coverage related to barriers due to socioeconomic factors (eg, lack of paid maternity leave, paid time off for health care visits) or disabilities (eg, movement disorders, vision loss, hearing loss). When enrolled as part of a larger study, subgroup-specific data for these various subpopulations should be adequately analyzed and reported.

We urge researchers working on future studies to evaluate and report outcomes that were not adequately reported in the identified evidence, such as adherence to condition-specific screening or testing, transition to care by primary care practitioners, maternal mortality, patient-reported outcomes (eg, quality of life), reduction in health inequities, worsening health inequities, and reported discrimination.

### Strengths and Limitations

We followed contemporary standards for systematic reviews, including (1) engagement with multiple types of stakeholders in defining and refining the research questions and (2) careful adherence to current systematic review standards for protocol publication and registration, literature searching, screening, data extraction, risk of bias assessment, qualitative synthesis, and SoE assessment. To maximize the applicability of the evidence to the US decision-making context, we restricted to US-based studies. The racial diversity of study participants generally mirrored the postpartum population in the US. On average, across the studies, patients ranged in age from their mid 20s to their mid 30s. As such, the conclusions in this systematic review apply generally to postpartum individuals in the US.

A few limitations of the evidence base are worth noting. Despite finding 28 studies, due to limited evidence for most outcomes of interest, we were able to make conclusions for only 2 prioritized outcomes, postpartum visit attendance and unplanned care utilization. Many of the prioritized outcomes were either not reported in any included study for specific comparisons or were reported in an insufficient number of studies to merit conclusions according to sufficient evidence. Some of these outcomes may be challenging for researchers to ascertain in retrospective studies because medical records may be incomplete if participants seek care at other clinical sites (eg, other hospitals or in other states) or if their insurance coverage changes following pregnancy or childbirth. In addition, harms were inadequately described; no study provided data for worsening health inequities or reported discrimination. The heterogeneity across studies in terms of the evaluated policy changes and comparisons also presented a challenge to our conducting a synthesis. We described the evidence narratively but recognize that differences in how “more comprehensive” insurance coverage was defined across studies varied.

## Conclusions

The findings of this systematic review suggest that more comprehensive health insurance coverage is probably associated with greater attendance at postpartum visits and may be associated with fewer preventable readmissions and emergency department visits, but the association of insurance coverage with other health care utilization, clinical, and harm outcomes is unclear. Future research should evaluate the impact of more comprehensive or extended health insurance on health outcomes in the postpartum period and beyond. Researchers should report separate data for various population subgroups, so that decision-makers can understand the implications of health insurance extension for different populations.

## Notes

Supplement 1. **eAppendix 1. **Search Strategies **eAppendix 2. **Summary of Design and Group Details **eAppendix 3. **Summary of Sample Details **eAppendix 4. **Risk of Bias Assessment—Confounding and Selection Bias **eAppendix 5. **Risk of Bias Assessment—Assessment of Remaining Biases, Quality, and Overall Risk of Bias **eAppendix 6. **Continuous Outcomes, Healthcare Utilization **eAppendix 7. **Categorical Outcomes, Healthcare Utilization **eAppendix 8. **Categorical Outcomes, Mental Health Symptoms

Supplement 2.
Data Sharing Statement

## References

[1] Petersen EE, Davis NL, Goodman D, . Racial/ethnic disparities in pregnancy-related deaths—United States, 2007-2016. MMWR Morb Mortal Wkly Rep. 2019;68(35):762-765. doi:10.15585/mmwr.mm6835a3 31487273 PMC6730892

[2] Centers for Disease Control and Prevention National Center for Health Statistics. Maternal mortality rates in the United States. 2022. Accessed October 25, 2022. https://www.cdc.gov/nchs/data/hestat/maternal-mortality/2020/maternal-mortality-rates-2020.htm

[3] Howell EA. Reducing disparities in severe maternal morbidity and mortality. Clin Obstet Gynecol. 2018;61(2):387-399. doi:10.1097/GRF.0000000000000349 29346121 PMC5915910

[4] Trost S, Beauregard J, Chandra G, . Pregnancy-related deaths: data from maternal mortality review committees in 36 US States, 2017–2019. Centers for Disease Control and Prevention. Accessed January 14, 2023. https://www.cdc.gov/reproductivehealth/maternal-mortality/erase-mm/data-mmrc.html

[5] Hardeman RR, Kheyfets A, Mantha AB, . Developing tools to report racism in maternal health for the CDC Maternal Mortality Review Information Application (MMRIA): findings from the MMRIA racism and discrimination working group. Matern Child Health J. 2022;26(4):661-669. doi:10.1007/s10995-021-03284-3 34982327

[6] Fabiyi CA, Reid LD, Mistry KB. Postpartum health care use after gestational diabetes and hypertensive disorders of pregnancy. J Womens Health (Larchmt). 2019;28(8):1116-1123. doi:10.1089/jwh.2018.7198 30628865

[7] Herrick CJ, Keller MR, Trolard AM, Cooper BP, Olsen MA, Colditz GA. Postpartum diabetes screening among low income women with gestational diabetes in Missouri 2010-2015. BMC Public Health. 2019;19(1):148. doi:10.1186/s12889-019-6475-0 30717710 PMC6360751

[8] Rodin D, Silow-Carroll S, Cross-Barnet C, Courtot B, Hill I. Strategies to promote postpartum visit attendance among medicaid participants. J Womens Health (Larchmt). 2019;28(9):1246-1253. doi:10.1089/jwh.2018.7568 31259648

[9] Thiel de Bocanegra H, Braughton M, Bradsberry M, Howell M, Logan J, Schwarz EB. Racial and ethnic disparities in postpartum care and contraception in California’s Medicaid program. Am J Obstet Gynecol. 2017;217(1):47.e1-47.e7. doi:10.1016/j.ajog.2017.02.040 28263752

[10] Health Care Payment Learning and Action Network (HCP LAN). Clinical episode payment models: maternity care. Accessed August 18, 2021. http://hcp-lan.org/workproducts/maternity-whitepaper-final.pdf

[11] Centers for Medicare and Medicaid Services. Lessons learned about payment strategies to improve postpartum care in Medicaid and CHIP. Accessed August 18, 2021. https://www.medicaid.gov/medicaid/quality-of-care/downloads/postpartum-payment-strategies.pdf

[12] Applegate M, Gee RE, Martin JN Jr. Improving maternal and infant health outcomes in Medicaid and the Children’s Health Insurance Program. Obstet Gynecol. 2014;124(1):143-149. doi:10.1097/AOG.0000000000000320 24901270

[13] Social Security Administration. Title XIX—grants for states for medical assistance programs. Accessed May 1, 2023. https://www.ssa.gov/OP_Home/ssact/title19/1900.htm

[14] 17th Congress (2021-2022). H.R.1319 - American Rescue Plan Act of 2021. Accessed July 28, 2022. https://www.congress.gov/bill/117th-congress/house-bill/1319

[15] Kaiser Family Foundation. Medicaid postpartum coverage extension tracker. Accessed February 26, 2023. https://www.kff.org/medicaid/issue-brief/medicaid-postpartum-coverage-extension-tracker/

[16] Berkman ND, Lohr KN, Ansari M, . AHRQ Methods for Effective Health Care Grading the Strength of a Body of Evidence When Assessing Health Care Interventions for the Effective Health Care Program of the Agency for Healthcare Research and Quality: An Update. Agency for Healthcare Research and Quality; 2008. 24404627

[17] abstrackr. Accessed May 5, 2023. http://abstrackr.cebm.brown.edu/account/login

[18] Agency for Healthcare Research and Quality. Systematic Review Data Repository. Accessed May 5, 2023. https://srdrplus.ahrq.gov/

[19] Higgins JP, Altman DG, Gøtzsche PC, ; Cochrane Bias Methods Group; Cochrane Statistical Methods Group. The Cochrane Collaboration’s tool for assessing risk of bias in randomised trials. BMJ. 2011;343:d5928. doi:10.1136/bmj.d5928 22008217 PMC3196245

[20] Sterne JA, Hernán MA, Reeves BC, . ROBINS-I: a tool for assessing risk of bias in non-randomised studies of interventions. BMJ. 2016;355:i4919. doi:10.1136/bmj.i4919 27733354 PMC5062054

[21] Berkman ND, Lohr KN, Ansari MT, . Grading the strength of a body of evidence when assessing health care interventions: an EPC update. J Clin Epidemiol. 2015;68(11):1312-1324. doi:10.1016/j.jclinepi.2014.11.023 25721570

[22] Gerrity M, Fiordalisi C, Pillay J, . AHRQ Methods for Effective Health Care. Roadmap for Narratively Describing Effects of Interventions in Systematic Reviews. Agency for Healthcare Research and Quality; 2020. doi:10.23970/AHRQEPCWHITEPAPERNARRATIVELY 33180401

[23] Murad MH, Fiordalisi C, Pillay J, . Making narrative statements to describe treatment effects. J Gen Intern Med. 2021;36(1):196-199. doi:10.1007/s11606-020-06330-y 33111244 PMC7858734

[24] Smith M, McCool-Myers M, Kottke MJ. Analysis of postpartum uptake of long-acting reversible contraceptives before and after implementation of Medicaid reimbursement policy. Matern Child Health J. 2021;25(9):1361-1368. doi:10.1007/s10995-021-03180-w 34109490

[25] Kozhimannil KB, Huskamp HA, Graves AJ, Soumerai SB, Ross-Degnan D, Wharam JF. High-deductible health plans and costs and utilization of maternity care. Am J Manag Care. 2011;17(1):e17-e25. 21485419

[26] Redd SK, Hall KS. Medicaid family planning expansions: the effect of state plan amendments on postpartum contraceptive use. J Womens Health (Larchmt). 2019;28(4):551-559. doi:10.1089/jwh.2018.7129 30484739 PMC6482903

[27] Rodriguez MI, Edelman A, Wallace N, Jensen JT. Denying postpartum sterilization to women with Emergency Medicaid does not reduce hospital charges. Contraception. 2008;78(3):232-236. doi:10.1016/j.contraception.2008.04.006 18692614

[28] Margerison CE, Hettinger K, Kaestner R, Goldman-Mellor S, Gartner D. Medicaid expansion associated with some improvements in perinatal mental health. Health Aff (Millwood). 2021;40(10):1605-1611. doi:10.1377/hlthaff.2021.00776 34606358 PMC9007181

[29] Kramer RD, Gangnon RE, Burns ME. Provision of immediate postpartum long-acting reversible contraceptives before and after Wisconsin Medicaid’s payment change. Womens Health Issues. 2021;31(4):317-323. doi:10.1016/j.whi.2021.02.009 33849768

[30] Myerson R, Crawford S, Wherry LR. Medicaid expansion increased preconception health counseling, folic acid intake, and postpartum contraception. Health Aff (Millwood). 2020;39(11):1883-1890. doi:10.1377/hlthaff.2020.00106 33136489 PMC7688246

[31] Taylor YJ, Liu TL, Howell EA. Insurance differences in preventive care use and adverse birth outcomes among pregnant women in a Medicaid nonexpansion state: a retrospective cohort study. J Womens Health (Larchmt). 2020;29(1):29-37. doi:10.1089/jwh.2019.7658 31397625 PMC6983742

[32] Brant AR, Kollikonda S, Yao M, Mei L, Emery J. Use of immediate postpartum long-acting reversible contraception before and after a state policy mandated inpatient access. Obstet Gynecol. 2021;138(5):732-737. doi:10.1097/AOG.0000000000004560 34619694

[33] Dunlop AL, Joski P, Strahan AE, Sierra E, Adams EK. Postpartum Medicaid coverage and contraceptive use before and after Ohio’s Medicaid expansion under the Affordable Care Act. Womens Health Issues. 2020;30(6):426-435. doi:10.1016/j.whi.2020.08.006 32958368

[34] Gordon SH, Sommers BD, Wilson IB, Trivedi AN. Effects of Medicaid expansion on postpartum coverage and outpatient utilization. Health Aff (Millwood). 2020;39(1):77-84. doi:10.1377/hlthaff.2019.00547 31905073 PMC7926836

[35] Arora KS, Wilkinson B, Verbus E, . Medicaid and fulfillment of desired postpartum sterilization. Contraception. 2018;97(6):559-564. doi:10.1016/j.contraception.2018.02.012 29490290 PMC5963995

[36] Schuster ALR, Perraillon MC, Paul JJ, Leiferman JA, Battaglia C, Morrato EH. The effect of the Affordable Care Act on women’s postpartum insurance and depression in 5 states that did not expand Medicaid, 2012-2015. Med Care. 2022;60(1):22-28. doi:10.1097/MLR.0000000000001652 34670222 PMC8811754

[37] Okoroh EM, Kane DJ, Gee RE, . Policy change is not enough: engaging provider champions on immediate postpartum contraception. Am J Obstet Gynecol. 2018;218(6):590.e1-590.e7. doi:10.1016/j.ajog.2018.03.007 29530670 PMC5970075

[38] Cilenti D, Kum HC, Wells R, Whitmire JT, Goyal RK, Hillemeier MM. Changes in North Carolina maternal health service use and outcomes among medicaid-enrolled pregnant women during state budget cuts. J Public Health Manag Pract. 2015;21(2):208-213. doi:10.1097/PHH.0000000000000118 25627330

[39] Steenland MW, Pace LE, Sinaiko AD, Cohen JL. Medicaid payments for immediate postpartum long-acting reversible contraception: evidence from South Carolina. Health Aff (Millwood). 2021;40(2):334-342. doi:10.1377/hlthaff.2020.00254 33523747 PMC9555010

[40] Steenland MW, Pace LE, Sinaiko AD, Cohen JL. Association between South Carolina Medicaid’s change in payment for immediate postpartum long-acting reversible contraception and birth intervals. JAMA. 2019;322(1):76-78. doi:10.1001/jama.2019.6854 31158852 PMC6547092

[41] Steenland MW, Wilson IB, Matteson KA, Trivedi AN. Association of Medicaid expansion in Arkansas with postpartum coverage, outpatient care, and racial disparities. JAMA Health Forum. 2021;2(12):e214167. doi:10.1001/jamahealthforum.2021.4167 35977301 PMC8796925

[42] Symum H, Zayas-Castro J. Impact of Statewide Mandatory Medicaid Managed Care (SMMC) programs on hospital obstetric outcomes. Healthcare (Basel). 2022;10(5):874. doi:10.3390/healthcare10050874 35628011 PMC9141169

[43] Wang X, Pengetnze YM, Eckert E, Keever G, Chowdhry V. Extending postpartum Medicaid beyond 60 days improves care access and uncovers unmet needs in a Texas Medicaid health maintenance organization. Front Public Health. 2022;10:841832. doi:10.3389/fpubh.2022.841832 35592081 PMC9110670

[44] Koch SK, Paul R, Addante AN, . Medicaid reimbursement program for immediate postpartum long-acting reversible contraception improves uptake regardless of insurance status. Contraception. 2022;113:57-61. doi:10.1016/j.contraception.2022.05.007 35588793

[45] Caudillo ML, Hurtado-Acuna C, Rendall MS, Boudreaux M. Association of the Delaware contraceptive access now initiative with postpartum LARC use. Matern Child Health J. 2022;26(8):1657-1666. doi:10.1007/s10995-022-03433-2 35488950 PMC9055365

[46] Eliason EL, Spishak-Thomas A, Steenland MW. Association of the affordable care act Medicaid expansions with postpartum contraceptive use and early postpartum pregnancy. Contraception. 2022;113:42-48. doi:10.1016/j.contraception.2022.02.012 35259409 PMC9378469

[47] Austin AE, Sokol RL, Rowland C. Medicaid expansion and postpartum depressive symptoms: evidence from the 2009-2018 Pregnancy Risk Assessment Monitoring System survey. Ann Epidemiol. 2022;68:9-15. doi:10.1016/j.annepidem.2021.12.011 34974107

[48] Rodriguez MI, Skye M, Lindner S, . Analysis of contraceptive use among immigrant women following expansion of Medicaid coverage for postpartum care. JAMA Netw Open. 2021;4(12):e2138983. doi:10.1001/jamanetworkopen.2021.38983 34910148 PMC8674744

[49] Pace LE, Saran I, Hawkins SS. Impact of Medicaid eligibility changes on long-acting reversible contraception use in Massachusetts and Maine. Med Care. 2022;60(2):119-124. doi:10.1097/MLR.0000000000001666 34908011

[50] Eliason EL, Daw JR, Allen HL. Association of Medicaid vs marketplace eligibility on maternal coverage and access with prenatal and postpartum care. JAMA Netw Open. 2021;4(12):e2137383. doi:10.1001/jamanetworkopen.2021.37383 34870677 PMC8649838

[51] DeSisto CL, Rohan A, Handler A, Awadalla SS, Johnson T, Rankin K. The effect of continuous versus pregnancy-only Medicaid eligibility on routine postpartum care in Wisconsin, 2011-2015. Matern Child Health J. 2020;24(9):1138-1150. doi:10.1007/s10995-020-02924-4 32335806

[52] Liberty A, Yee K, Darney BG, Lopez-Defede A, Rodriguez MI. Coverage of immediate postpartum long-acting reversible contraception has improved birth intervals for at-risk populations. Am J Obstet Gynecol. 2020;222(4S):S886.e1-S886.e9. doi:10.1016/j.ajog.2019.11.1282 PMC7147501 31846612

[53] Steenland MW, Wherry LR. Medicaid expansion led to reductions in postpartum hospitalizations. Health Aff (Millwood). 2023;42(1):18-25. doi:10.1377/hlthaff.2022.00819 36623214 PMC10882633

