# Supplementary material for: Health Insurance Coverage and Postpartum Outcomes in the US: A Systematic Review
Source: JAMA Netw Open. 2023 Jun 2;6(6):e2316536. doi: 10.1001/jamanetworkopen.2023.16536 (PMC10238947; doi:10.1001/jamanetworkopen.2023.16536)
Supplement: Supplement 1. — eAppendix 1. Search Strategies eAppendix 2. Summary of Design and Group Details eAppendix 3. Summary of Sample Details eAppendix 4. Risk of Bias Assessment—Confounding and Selection Bias eAppendix 5. Risk of Bias Assessment—Assessment of Remaining Biases, Quality, and Overall Risk of Bias eAppendix 6. Continuous Outcomes, Healthcare Utilization eAppendix 7. Categorical Outcomes, Healthcare Utilization eAppendix 8. Categorical Outcomes, Mental Health Symptoms [file jamanetwopen-e2316536-s001.pdf]

# Supplemental Online Content

Saldanha IJ, Adam GP, Kanaan G, et al. Health insurance coverage and postpartum outcomes in the US: a systematic review. *JAMA Netw Open*. 2023;6(6):e2316536. doi:10.1001/jamanetworkopen.2023.16536

**eAppendix 1.** Search Strategies
**eAppendix 2.** Summary of Design and Group Details
**eAppendix 3.** Summary of Sample Details
**eAppendix 4.** Risk of Bias Assessment—Confounding and Selection Bias
**eAppendix 5.** Risk of Bias Assessment—Assessment of Remaining Biases, Quality, and Overall Risk of Bias
**eAppendix 6.** Continuous Outcomes, Healthcare Utilization
**eAppendix 7.** Categorical Outcomes, Healthcare Utilization
**eAppendix 8.** Categorical Outcomes, Mental Health Symptoms

This supplemental material has been provided by the authors to give readers additional information about their work.

© 2023 Saldanha IJ et al. *JAMA Network Open.*

**eAppendix 1. Search Strategies**

**Medline** (via **PubMed**)

(((((postpartum[tiab] OR post-partum[tiab] OR postnatal[tiab] OR post-natal[tiab] OR puerperal[tiab] OR puerperium[tiab] OR postdelivery[tiab] OR post-delivery[tiab] OR "post delivery"[tiab] OR "Peripartum Period"[Mesh] OR "fourth trimester"[tiab] OR "4th trimester"[tiab]) AND ("Perinatal Care"[Mesh] OR "Postnatal Care"[Mesh] OR "care coordination"[tiab] OR "care co-ordination" [tiab] OR "coordination of care"[tiab] OR "co-ordination of care"[tiab] OR "Case Management"[Mesh] OR "Coordinated care"[tiab] OR "Co-ordinated care"[tiab] OR "collaborative care"[tiab] OR "integrated care"[tiab] OR "shared care"[tiab] OR "transitional care"[tiab] OR "comanagement"[tiab] OR "co-management"[tiab] OR "case management"[tiab] OR "multidisciplinary care"[tiab] OR "interdisciplinary care"[tiab] OR "disease management"[tiab] OR "Progressive Patient Care"[MeSH] OR "Continuity of Patient Care"[MeSH] OR "Patient-Centered Care"[MeSH] OR "Patient Care Planning"[MeSH] OR "Disease Management"[MeSH] OR "Delivery of Health Care, Integrated"[MeSH] OR "Delivery of Health Care "[Mesh] OR "Standard of Care"[Mesh] OR "Health Services Accessibility"[Mesh] OR "Appointments and Schedules"[Mesh] OR ((care OR treatment) AND (timing OR integrat* OR collaborat* OR coordinat* OR transition* OR interdisciplin* OR shared OR comanagement OR cooperat* OR aftercare OR interinstitution* OR synchron* OR harmon* OR manage*)) OR "Office Visits"[Mesh] OR "office visits"[tiab] OR Doula[tiab] OR Midwife[tiab] OR "Home visit*"[tiab] OR "home-visit*"[tiab] OR "group visit"[tiab] OR "support group"[tiab] OR centering[tiab] OR holistic[tiab] OR (("Lay support" OR Outreach OR "community health") AND worker) OR "Patient navigator"[tiab] OR "Telemedicine"[Mesh] OR "Remote Consultation"[Mesh] OR ehealth[tiab] OR e-health*[tiab] OR mhealth*[tiab] OR m-health*[tiab] OR telemedicine[tiab] OR telehealth[tiab] OR telecare[tiab] OR smartphone*[tiab] OR "smart phone*"[tiab] OR "smart-phone*"[tiab] OR "cell phone*"[tiab] OR "mobile phone*"[tiab] OR ((remote* OR video OR virtual OR phone) AND (care OR consult* OR visit* OR service*)) OR "remote monitoring"[tiab] OR Telemonitoring[tiab] OR wearable[tiab] OR wireless[tiab] OR portable[tiab] OR "Health education"[tiab] OR Contracept*[tiab] OR insurance expansion[tiab] OR Medicaid expansion[tiab] OR "Maternal Death/prevention and control"[MESH] OR "Maternal-Child Health Services"[Mesh]))) NOT ((("Africa"[Mesh] OR "Algeria"[Mesh] OR "Angola"[Mesh] OR "Bangladesh"[Mesh] OR "Benin"[Mesh] OR "Bhutan"[Mesh] OR "Bolivia"[Mesh] OR "Cabo Verde"[Mesh] OR "Cambodia"[Mesh] OR "Cameroon"[Mesh] OR "Comoros"[Mesh] OR "Cote d'Ivoire"[Mesh] OR "Democratic Republic of the Congo"[Mesh] OR "Djibouti"[Mesh] OR "Egypt"[Mesh] OR "El Salvador"[Mesh] OR "Eswatini"[Mesh] OR "Ghana"[Mesh] OR "Honduras"[Mesh] OR "India"[Mesh] OR "Kenya"[Mesh] OR "Kyrgyzstan"[Mesh] OR "Laos"[Mesh] OR "Lesotho"[Mesh] OR "Mauritania"[Mesh] OR "Micronesia"[Mesh] OR "Moldova"[Mesh] OR "Mongolia"[Mesh] OR "Morocco"[Mesh] OR "Myanmar"[Mesh] OR "Nepal"[Mesh] OR "Nicaragua"[Mesh] OR "Nigeria"[Mesh] OR "Pakistan"[Mesh] OR "Papua New Guinea"[Mesh] OR "Philippines"[Mesh] OR "Sao Tome and Principe"[Mesh] OR "Senegal"[Mesh] OR "Sri Lanka"[Mesh] OR "Tanzania"[Mesh] OR "Timor-Leste"[Mesh] OR "Tunisia"[Mesh] OR "Ukraine"[Mesh] OR "Uzbekistan"[Mesh] OR "Vanuatu"[Mesh] OR "Vietnam"[Mesh] OR "Zambia"[Mesh] OR "Zimbabwe"[Mesh] OR "Afghanistan"[Mesh] OR "Burundi"[Mesh] OR "Burkina Faso"[Mesh] OR "Central African Republic"[Mesh] OR "Eritrea"[Mesh] OR "Ethiopia"[Mesh] OR "Guinea"[Mesh] OR "Gambia"[Mesh] OR "Guinea-Bissau"[Mesh] OR "Haiti"[Mesh] OR "Liberia"[Mesh] OR

© 2023 Saldanha IJ et al. *JAMA Network Open.*

"Madagascar"[Mesh] OR "Mali"[Mesh] OR "Mozambique"[Mesh] OR "Malawi"[Mesh] OR "Niger"[Mesh] OR "Democratic People's Republic of Korea"[Mesh] OR "Rwanda"[Mesh] OR "Sudan"[Mesh] OR "Sierra Leone"[Mesh] OR "Somalia"[Mesh] OR "South Sudan"[Mesh] OR "Syria"[Mesh] OR "Chad"[Mesh] OR "Togo"[Mesh] OR "Tajikistan"[Mesh] OR "Uganda"[Mesh] OR "Yemen"[Mesh] OR Somaliland OR "Albania"[Mesh] OR "Argentina"[Mesh] OR "Armenia"[Mesh] OR "American Samoa"[Mesh] OR "Azerbaijan"[Mesh] OR "Bulgaria"[Mesh] OR "Bosnia and Herzegovina"[Mesh] OR "Republic of Belarus"[Mesh] OR "Belize"[Mesh] OR "Brazil"[Mesh] OR "Botswana"[Mesh] OR "China"[Mesh] OR "Colombia"[Mesh] OR "Costa Rica"[Mesh] OR "Cuba"[Mesh] OR "Dominica"[Mesh] OR "Dominican Republic"[Mesh] OR "Ecuador"[Mesh] OR "Fiji"[Mesh] OR "Gabon"[Mesh] OR "Georgia (Republic)"[Mesh] OR "Equatorial Guinea"[Mesh] OR "Grenada"[Mesh] OR "Guatemala"[Mesh] OR "Guyana"[Mesh] OR "Indonesia"[Mesh] OR "Iran"[Mesh] OR "Iraq"[Mesh] OR "Jamaica"[Mesh] OR "Jordan"[Mesh] OR "Kazakhstan"[Mesh] OR "Lebanon"[Mesh] OR "Libya"[Mesh] OR "Saint Lucia"[Mesh] OR "Indian Ocean Islands"[Mesh] OR "Mexico"[Mesh] OR "Micronesia"[Mesh] OR "Republic of North Macedonia"[Mesh] OR "Montenegro"[Mesh] OR "Malaysia"[Mesh] OR "Namibia"[Mesh] OR "Peru"[Mesh] OR "Paraguay"[Mesh] OR "Russia"[Mesh] OR "Serbia"[Mesh] OR "Suriname"[Mesh] OR "Thailand"[Mesh] OR "Turkmenistan"[Mesh] OR "Tonga"[Mesh] OR "Turkey"[Mesh] OR "Saint Vincent and the Grenadines"[Mesh] OR "Venezuela"[Mesh] OR "Samoa"[Mesh] OR "Kosovo"[Mesh] OR "South Africa"[Mesh]) NOT ("Aruba"[Mesh] OR "Andorra"[Mesh] OR "United Arab Emirates"[Mesh] OR "Antigua and Barbuda"[Mesh] OR "Australia"[Mesh] OR "Austria"[Mesh] OR "Belgium"[Mesh] OR "Bahrain"[Mesh] OR "Bahamas"[Mesh] OR "Bermuda"[Mesh] OR "Barbados"[Mesh] OR "Brunei"[Mesh] OR "Canada"[Mesh] OR "Switzerland"[Mesh] OR "Channel Islands"[Mesh] OR "Chile"[Mesh] OR "Curacao"[Mesh] OR "Cyprus"[Mesh] OR "Czech Republic"[Mesh] OR "Germany"[Mesh] OR "Denmark"[Mesh] OR "Spain"[Mesh] OR "Estonia"[Mesh] OR "Finland"[Mesh] OR "France"[Mesh] OR "United Kingdom"[Mesh] OR "Gibraltar"[Mesh] OR "Greece"[Mesh] OR "Greenland"[Mesh] OR "Guam"[Mesh] OR "Hong Kong"[Mesh] OR "Croatia"[Mesh] OR "Hungary"[Mesh] OR "Ireland"[Mesh] OR "Iceland"[Mesh] OR "Israel"[Mesh] OR "Italy"[Mesh] OR "Japan"[Mesh] OR "Republic of Korea"[Mesh] OR "Kuwait"[Mesh] OR "Liechtenstein"[Mesh] OR "Lithuania"[Mesh] OR "Luxembourg"[Mesh] OR "Latvia"[Mesh] OR "Macau"[Mesh] OR "Monaco"[Mesh] OR "Malta"[Mesh] OR "Mauritius"[Mesh] OR "New Caledonia"[Mesh] OR "Netherlands"[Mesh] OR "Norway"[Mesh] OR "New Zealand"[Mesh] OR "Oman"[Mesh] OR "Panama"[Mesh] OR "Palau"[Mesh] OR "Poland"[Mesh] OR "Puerto Rico"[Mesh] OR "Portugal"[Mesh] OR "Qatar"[Mesh] OR "Romania"[Mesh] OR "Saudi Arabia"[Mesh] OR "Singapore"[Mesh] OR "San Marino"[Mesh] OR "Slovakia"[Mesh] OR "Slovenia"[Mesh] OR "Sweden"[Mesh] OR "Sint Maarten"[Mesh] OR "Seychelles"[Mesh] OR "Trinidad and Tobago"[Mesh] OR "Taiwan"[Mesh] OR "Uruguay"[Mesh] OR "United States"[Mesh] OR "British Virgin Islands"[Mesh] OR "United States Virgin Islands"[Mesh])))) NOT (("address"[pt] OR "autobiography"[pt] OR "bibliography"[pt] OR "biography"[pt] OR "congress"[pt] OR "dictionary"[pt] OR "directory"[pt] OR "festschrift"[pt] OR "government publication"[pt] OR "historical article"[pt] OR "interview"[pt] OR "case reports"[pt] OR "Cross-Sectional Studies"[Mesh] OR "Focus Groups"[Mesh] OR ("Review"[pt] NOT ("Systematic Review" OR "scoping review" OR "clinical trial" OR "Randomized Controlled Trial")) OR "lecture"[pt] OR "legal case"[pt] OR "legislation"[pt] OR "news"[pt] OR "newspaper article"[pt] OR "patient education handout"[pt] OR "periodical index"[pt] OR "in vitro techniques"[mh] OR "introductory journal article"[pt] OR "Editorial"[pt] OR ("Animals"[Mesh] NOT "Humans"[Mesh]) OR rats[tw] OR rat[tw] OR cow[tw] OR cows[tw] OR chicken*[tw] OR horse[tw] OR horses[tw] OR mice[tw] OR mouse[tw]

© 2023 Saldanha IJ et al. *JAMA Network Open.*

OR bovine[tw] OR sheep[tw] OR ovine[tw] OR murinae[tw] OR cats[tw] OR cat[tw] OR dog[tw] OR dogs[tw] OR rodent[tw]))) AND (("Cohort Studies"[Mesh] OR cohort OR "Clinical Trial" [Publication Type] OR (follow-up OR followup) OR "Insurance Coverage "[Mesh] OR "different models" OR longitudinal OR "Placebos"[Mesh] OR placebo* OR "Research Design"[Mesh] OR "Evaluation Study" [Publication Type] OR "Comparative Study" [Publication Type] OR ((comparative OR Intervention) AND study) OR pretest* OR posttest* OR prepost* OR "before and after" OR interrupted time* OR time serie* OR intervention* OR ((quasi-experiment* OR quasiexperiment* OR quasi OR experimental) AND (method OR study OR trial OR design*)) OR "real world" OR "real-world" OR "Case-Control Studies"[Mesh] OR (case AND control) OR "Random Allocation"[Mesh] OR "Clinical Trial" [Publication Type] OR "Double-Blind Method"[Mesh] OR "Single-Blind Method"[Mesh] OR random* OR "Placebos"[Mesh] OR placebo OR ((clinical OR controlled) AND trial*) OR ((singl* OR doubl* OR trebl* OR tripl*) AND (blind* OR mask*)) OR rct OR crossover OR cross-over OR cross-over OR "treatment switching" OR "Treatment Switching"[Mesh] OR RCT OR "Randomized Controlled Trial" [Publication Type]))

**EMBASE**

| No. | Query | Results |
|---|---|---|
| #6. | #3 AND #4 AND [article]/lim | |
| #5. | #3 AND #4 | |
| #4. | 'cohort studies'/exp OR longitudinal OR ((comparative OR intervention) AND study) OR prepost* OR 'before and after' OR 'interrupted time*' OR 'time serie*' OR intervention* OR (('quasi experiment*' OR quasiexperiment* OR quasi OR experimental) AND (method OR study OR trial OR design*)) OR 'real world' OR 'random allocation'/exp OR 'double-blind method'/exp OR 'single-blind method'/exp OR random* OR ((clinical OR controlled) AND trial*) | |
| #3. | #1 AND #2 | |
| #2. | 'care coordination' OR 'care co-ordination' OR 'coordination of care' OR 'co-ordination of care' OR 'case management'/exp OR 'case management' OR 'interdisciplinary care' OR 'continuity of patient care'/exp OR 'patient-centered care'/exp OR 'patient care planning'/exp OR ((care OR treatment) AND (timing OR integrat* OR collaborat* OR coordinat* OR transition* OR interdisciplin* OR shared OR comanagement OR cooperat* OR aftercare OR interinstitution* OR synchron* OR harmon* OR manage*)) OR 'home visit*' OR 'telemedicine'/exp OR 'remote consultation'/exp | |
| #1. | 'postpartum'/exp OR postpartum OR 'fourth trimester' OR '4th trimester' | |

© 2023 Saldanha IJ et al. *JAMA Network Open.*

## Cochrane CENTRAL

#1	postpartum OR post-partum OR postnatal OR post-natal OR postdelivery OR post-delivery OR "post delivery" OR "fourth trimester" OR "4th trimester"	16558
#2	"care coordination" OR "care co-ordination" OR "coordination of care" OR "co-ordination of care" OR [mh "Case Management"] OR "Coordinated care" OR "Co-ordinated care" OR "collaborative care" OR "integrated care" OR "shared care" OR "transitional care" OR comanagement OR co-management OR "case management" OR "interdisciplinary care" OR "disease management" OR [mh "Progressive Patient Care"] OR [mh "Continuity of Patient Care"] OR [mh "Patient-Centered Care"] OR [mh "Patient Care Planning"] OR ((care OR treatment ) AND (timing OR integrat* OR collaborat* OR coordinat* OR transition* OR interdisciplin* OR shared OR comanagement OR cooperat* OR aftercare OR interinstitution* OR synchron* OR harmon* OR manage* )) OR "Patient navigator" OR [mh Telemedicine] OR [mh "Remote Consultation"] OR ehealth OR e-health* OR mhealth* OR m-health* OR telemedicine OR telehealth OR "insurance expansion" OR "Medicaid expansion"	208303
#3	#1 AND #2

## CINAHL

((postpartum OR post-partum OR postnatal OR post-natal OR postdelivery OR post-delivery OR "post delivery" OR OR "fourth trimester" OR "4th trimester" ) AND ("care coordination" OR "care co-ordination" OR "coordination of care" OR "co-ordination of care" OR (MH "Case Management"+) OR "Coordinated care" OR "Co-ordinated care" OR "collaborative care" OR "integrated care" OR "shared care" OR "transitional care" OR comanagement OR co-management OR "case management" OR "interdisciplinary care" OR "disease management" OR (MH "Progressive Patient Care"+) OR (MH "Continuity of Patient Care"+) OR (MH "Patient-Centered Care"+) OR (MH "Patient Care Planning"+) OR (MH "Disease Management"+) OR (MH "Delivery of Health Care, Integrated"+) OR (MH "Delivery of Health Care"+) OR (MH "Standard of Care"+) OR (MH "Health Services Accessibility"+) OR (MH "Appointments and Schedules"+) OR ((care OR treatment ) AND (timing OR integrat* OR collaborat* OR coordinat* OR transition* OR interdisciplin* OR shared OR comanagement OR cooperat* OR aftercare OR interinstitution* OR synchron* OR harmon* OR manage* )) OR (MH "Office Visits"+) OR "office visits" OR Doula OR Midwife OR "Home visit*" OR home-visit* OR "group visit" OR "support group" OR centering OR holistic OR (("Lay support" OR Outreach OR "community health" ) AND worker ) OR "Patient navigator" OR (MH Telemedicine+) OR (MH "Remote Consultation"+) OR ehealth OR e-health* OR mhealth* OR m-health* OR telemedicine OR telehealth) OR "insurance expansion" OR "Medicaid expansion" OR (MH "Maternal Death/prevention and control"+) OR (MH "Maternal-Child Health Services"+))) AND ((MH "Cohort Studies"+) OR cohort OR PT "Clinical Trial" OR longitudinal OR PT "Evaluation Study" OR PT "Comparative Study" OR ((comparative OR Intervention ) AND study ) OR pretest* OR posttest* OR prepost* OR "before and after" OR "interrupted time*" OR "time serie*" OR intervention* OR ((quasi-experiment* OR quasiexperiment* OR quasi OR experimental ) AND (method OR study OR trial OR design* )) OR (MH "Random Allocation"+) OR PT "Clinical Trial" OR

© 2023 Saldanha IJ et al. *JAMA Network Open.*

(MH "Double-Blind Method"+) OR (MH "Single-Blind Method"+) OR random* OR PT "Randomized Controlled Trial")

**ClinicalTrials.gov**

(postpartum OR post-partum OR postnatal OR post-natal OR puerperal OR puerperium OR postdelivery OR post-delivery OR Peripartum OR Peri-partum)
AND
United States OR Canada

© 2023 Saldanha IJ et al. *JAMA Network Open.*

**eAppendix 2. Summary of Design and Group Details**

| Author, Year, PMID | State(s) | Funding (Study Years) | Over all RoB | Inclusion Criteria | Exclusion Criteria | Study N | Focus of Study | Arm | Arm N | Arm Description |
|---|---|---|---|---|---|---|---|---|---|---|
| Arora, 2018, 29490290 | OH | Non-industry (2012-2014) | High | Sterilization as the documented contraceptive plan | Sterilized before study or died | 1184 | Contraceptive care | More comprehensive insurance | 154 | Private insurance |
| | . | . | . | . | . | . | . | Less comprehensive insurance | 1030 | Medicaid insurance |
| Austin, 2022, 34974107 | 20 states | Non-industry (2009-2018) | Mod erate | Age ≥18 yr<br>Income <138% FPL | NR | 82728 | General PP care | More comprehensive insurance coverage | 51200 | Medicaid expansion states |
| | . | . | . | . | . | | . | Less comprehensive insurance coverage | 31528 | Medicaid non-expansion states |
| Brant, 2021, 34619694 | OH | Non-industry (2015-2019) | Mod erate | Gestational age ≥20 wk | Birth outcome not a liveborn neonate | 8516 | Contraceptive care | More comprehensive access to care | 2129 | Law that required hospitals to offer LARC placement after delivery (2017-2019) |
| | . | . | . | . | . | . | . | Less comprehensive access to care | 6387 | No law that required hospitals to offer after delivery (2015-2017) |
| Caudillo, 2022, 35488950 | 16 states | Non-industry (2012-2017) | Mod erate | NR | NR | 47109 | Contraceptive care | More comprehensive insurance | 2504 | Delaware (After Delaware Contraceptive Access Now (DelCAN) initiative) |
| | . | . | . | . | . | . | . | Less comprehensive insurance | 44605 | 15 other states (no Delaware Contraceptive Access Now (DelCAN) initiative) |
| Cilenti, 2015, 25627330 | NC | Non-industry (2009-2010) | Mod erate | Delivery covered by NC Medicaid | NR | 1969 | General PP care | More comprehensive insurance | 1007 | Before change in Medicaid policy reducing reimbursement rates for maternity care coordination by 19% |
| | | . | . | . | . | . | . | Less comprehensive insurance | 962 | After change in Medicaid policy reducing reimbursement rates for maternity care coordination by 19% |
| DeSisto, 2020, 32335806 | WI | Non-industry (2011-2015) | Mod erate | Live births. Medicaid. | NR | 105718 | General PP care | More comprehensive insurance | 79172 | Continuous Medicaid eligibility |

© 2023 Saldanha IJ et al. *JAMA Network Open.*

| Author, Year, PMID | State(s) | Funding (Study Years) | Overall RoB | Inclusion Criteria | Exclusion Criteria | Study N | Focus of Study | Arm | Arm N | Arm Description |
|---|---|---|---|---|---|---|---|---|---|---|
| | . | . | . | . | . | | . | Less comprehensive insurance | 26546 | Pregnancy-only Medicaid eligibility |
| Dunlop, 2020, 32958368 | OH | Non-industry (2011-2015) | Moderate | Age 20-44 yr Medicaid | Medicaid eligibility based on disability | 138426 | Contraceptive care | More comprehensive insurance | 54477 | After Medicaid expansion (2014-2015) |
| | . | . | . | . | . | . | . | Less comprehensive insurance | 83949 | Before Medicaid expansion (2011-2013) |
| Eliason, 2021, 34870677 | 15 states | Non-industry (2011-2018) | Moderate | Age >18 yr Income 100%-138% of FPL | NR | 5034 | General PP care | More comprehensive insurance | 3389 | Medicaid expansion states |
| | . | . | . | . | . | . | . | Less comprehensive insurance | 1645 | Medicaid non-expansion states |
| Eliason, 2022, 35259409 | 11 states | Non-industry (2012-2019) | Moderate | Age ≥18 yr Income ≤138% FPL | NR | 34598 | General PP care | More comprehensive insurance | 25781 | Medicaid expansion states |
| | . | . | . | . | . | . | . | Less comprehensive insurance | 8817 | Medicaid non-expansion states |
| Gordon, 2020, 31905073 | CO, UT | Non-industry (2013-2015) | Moderate | Age ≥19 yr Live birth | NR | 66672 | General PP care | More comprehensive insurance | 42144 | CO (after Medicaid expansion) |
| | . | . | . | . | . | . | . | Less comprehensive insurance | 24528 | UT (no Medicaid expansion) |
| Koch, 2022, 35588793 | MO | NR (2017-2019) | | Gestational age >24 wk | NR | 6233 | Contraceptive care | More comprehensive insurance | 3128 | After policy change for separate LARC reimbursement |
| | . | . | . | . | . | . | . | Less comprehensive insurance | 3105 | Before policy change for separate LARC reimbursement |
| Kozhimannil, 2011, 21485419 | MA | Non-industry (2001-2007) | Moderate | NR | NR | 2509 | General PP care | More comprehensive insurance | 2280 | Full coverage of AP and PP care, no cost sharing beyond office visit and hospitalization copayments. Out-patient visit copayments $5-$25 (median $15). Hospitalization copayments $0-$1000 (median $250). |

© 2023 Saldanha IJ et al. *JAMA Network Open.*

| Author, Year, PMID | State(s) | Funding (Study Years) | Overall RoB | Inclusion Criteria | Exclusion Criteria | Study N | Focus of Study | Arm | Arm N | Arm Description |
|---|---|---|---|---|---|---|---|---|---|---|
| | . | . | . | . | . | . | . | Less comprehensive insurance | 229 | Annual deductible $500-$2000 for individuals and $1000-$4000 for families. Out-of-pocket maximum $2000-$4000 for individuals and $4000-$8000 for families. |
| Kramer, 2021, 33849768 | WI | Non-industry (2016-2017) | Moderate | Overall/general PP/ pregnant population Medicaid | NR | 45200 | Contraceptive care | More comprehensive insurance | 22405 | After unbundling (separate or additional reimbursement for immediate PP LARC) |
| | . | . | . | . | . | . | . | Less comprehensive insurance | 22795 | Before unbundling (no separate or additional reimbursement for immediate PP LARC) |
| Liberty, 2020, 31846612 | SC | Non-industry (2010-2017) | Moderate | Gestational age ≥ 23 wk<br>Singleton pregnancy | Births covered by Emergency Medicaid | 164004 | Contraceptive care | More comprehensive insurance | 108430 | After policy covering immediate PP LARC (2013-2017) |
| | . | . | | . | . | . | . | Less comprehensive insurance | 55574 | Before policy covering immediate PP LARC (2010-2012) |
| Margerison, 2021, 34606358 | 18 states | Non-industry (2012-2018) | Moderate | Age ≥18 yr<br>Household income ≤137% of FPL | NR | 56965 | General/ Overall PP care | More comprehensive insurance | NR | Medicaid expansion states |
| | . | . | . | . | . | . | . | Less comprehensive insurance | NR | Medicaid non-expansion states |
| Myerson, 2020, 33136489 | 13 states | NR (2011-2017) | Moderate | Household income ≤138% of FPL<br>Live birth | NR | 15059 | Contraceptive care | More comprehensive insurance | 9135 | Medicaid expansion states |
| | . | . | . | . | . | . | . | Less comprehensive insurance | 5924 | Medicaid non-expansion states |
| Okoroh, 2018, 29530670 | IA, LA | NR (2013-2015) | Moderate | Medicaid | NR | 57894 | Contraceptive care | More comprehensive insurance | NR | After Medicaid expansion (2014-2015) |
| | . | . | . | . | . | . | . | Less comprehensive insurance | NR | Before Medicaid expansion (2013-2014) |
| Pace, 2022, 34908011 | MA, ME | Non-industry (2009-2015) | High | Age 13-45 yr<br>Medicaid insurance | NR | 776853 | General PP care | More comprehensive insurance | 691867 | Massachusetts (after Medicaid expansion) |

© 2023 Saldanha IJ et al. *JAMA Network Open.*

| Author, Year, PMID | State(s) | Funding (Study Years) | Overall RoB | Inclusion Criteria | Exclusion Criteria | Study N | Focus of Study | Arm | Arm N | Arm Description |
|---|---|---|---|---|---|---|---|---|---|---|
| | . | . | | . | . | | . | Less comprehensive insurance | 84986 | Maine (after Medicaid contraction) |
| Redd, 2019, 30484739 | OK, WI, MD, MN, MO, NY, OR, PA, WA | Non-industry (2007-2013) | Moderate | Live birth | NR | 75082 | Contraceptive care | More comprehensive insurance | 19882 | Transition from the Medicaid 1115 waiver, which allowed states to expand eligibility to some individuals otherwise ineligible Medicaid coverage, to the State Plan Amendment, which provides contraceptive care to all |
| | . | . | . | . | . | . | . | Less comprehensive insurance | 55200 | Maintenance of the Medicaid 1115 waiver, which allowed states to expand eligibility to some individuals otherwise ineligible Medicaid coverage |
| Rodriguez, 2008, 18692614 | OR | NR (2000-2006) | Moderate | Convenience sample based on available billing data | NR | 11526 | Contraceptive care | More comprehensive insurance | 7832 | Before policy requiring undocumented immigrants and legal immigrants within 5 years of immigration with Emergency Medicaid to pay for sterilization following vaginal delivery |
| | . | . | . | . | . | . | . | Less comprehensive insurance | 3694 | After policy requiring undocumented immigrants and legal immigrants within 5 years of immigration with Emergency Medicaid to pay for sterilization following vaginal delivery |
| Rodriguez, 2021, 34910148 | OR, SC | Non-industry (2014-2019) | Moderate | Age 15-44 yr Low-income, noncitizen, Emergency Medicaid. | LARC | 27667 | General PP care | More comprehensive insurance | 15465 | Oregon (after Medicaid expansion) |
| | . | . | . | . | . | . | . | Less comprehensive insurance | 12202 | South Carolina (no Medicaid expansion) |
| Schuster, 2022, 34670222 | MO, NE, OK, UT, WY | Non-industry (2012-2015) | Moderate | Family income <100% or >400% of FPL | Uninsured for pregnancy | 9472 | General/ Overall PP care | More comprehensive insurance | 4797 | After Medicaid expansion (2014-2015) |

© 2023 Saldanha IJ et al. *JAMA Network Open.*

| Author, Year, PMID | State(s) | Funding (Study Years) | Overall RoB | Inclusion Criteria | Exclusion Criteria | Study N | Focus of Study | Arm | Arm N | Arm Description |
|---|---|---|---|---|---|---|---|---|---|---|
| | . | . | . | . | . | . | . | Less comprehensive insurance | 4675 | Before Medicaid expansion (2012-2013) |
| Smith, 2021, 34109490 | GA | NR (2015-2017) | Moderate | Age ≤44 yr | NR | 5648 | Contraceptive care | More comprehensive insurance | 3683 | After Medicaid policy covering inpatient LARC (2016-2017) |
| | . | . | . | . | . | . | . | Less comprehensive insurance | 1965 | Before Medicaid policy covering inpatient LARC (2015) |
| Steenland, 2021a, 33523747 | SC | Non-industry (2010-2014) | Moderate | Age 12-50 yr<br>Births covered by South Carolina Medicaid | NR | 154163 | Contraceptive care | More comprehensive insurance | NR | After Medicaid policy of payment for immediate PP LARC (Feb 2012-2014) |
| | . | . | . | . | . | . | . | Less comprehensive insurance | NR | Before Medicaid policy of payment for immediate PP LARC (2011-Jan 2012) |
| Steenland, 2021b, 35977301 | AR | Industry (2013-2015) | High | Age ≥19 yr | NR | 50364 | General PP care | More comprehensive insurance | 40785 | After Medicaid expansion (2014-2015) |
| | . | . | . | . | | | . | Less comprehensive insurance | 9579 | Before Medicaid expansion (2013) |
| Symum, 2022, 35628011 | FL | Not funded (2010-2017) | Moderate | Hospital delivery | NR | 1454699 | General PP care | More comprehensive insurance | 662981 | After Statewide Mandatory Medicaid Managed Care (2014-2017) |
| | . | . | . | . | . | . | . | Less comprehensive insurance | 791718 | Before Statewide Mandatory Medicaid Managed Care (2010-2014) |
| Taylor, 2020, 31397625 | NC | Non-industry (2014-2015) | Moderate | Age ≥18 yr<br>Gestational age ≤42 wk<br>Live birth | Insurance not commercial, Medicaid, or uninsured | 9613 | General/ Overall PP care | Commercial insurance | 4441 | NR |
| | . | . | . | . | . | . | . | Medicaid insurance | 4990 | NR |
| | . | . | . | . | . | . | . | No insurance | 182 | NR |
| Wang, 2022, 35592081 | TX | NR (2019-2020) | High | Age 14-48 yr<br>Singleton pregnancy | NR | 8876 | General PP care | More comprehensive insurance | 5411 | After Families First Coronavirus Response Act (2020) |

© 2023 Saldanha IJ et al. *JAMA Network Open.*

| Author, Year, PMID | State(s) | Funding (Study Years) | Over all RoB | Inclusion Criteria | Exclusion Criteria | Study N | Focus of Study | Arm | Arm N | Arm Description |
|---|---|---|---|---|---|---|---|---|---|---|
| | . | . | . | . | . | . | . | Less comprehensive insurance | 3465 | Before Families First Coronavirus Response Act (2019) |

Abbreviations: AP = antepartum, FPL = federal poverty limit, LARC = long-acting reversible contraception, NR = not reported, PMID = PubMed ID, PP = postpartum, RoB = risk of bias

All studies were retrospective nonrandomized comparative studies (NRCSs).

© 2023 Saldanha IJ et al. *JAMA Network Open.*

**eAppendix 3. Summary of Sample Details**

| Author, Year, PMID | Age in Years, Mean (SD) or as Specified | BMI, Mean (SD) or as Specified | Race, N (%) | Educational Attainment in Years, Mean (SD) or as Specified | Employment Status | SES | Miscellaneous N (%) | Chronic Conditions | Delivery Type | Offspring Characteristics |
|---|---|---|---|---|---|---|---|---|---|---|
| Arora, 2018, 29490290 | 30 (5.4) | NR | W: 363 (30.7)<br>B: 592 (50.0)<br>A: 10 (0.9)<br>H: 186 (15.7)<br>O: 33 (2.8) | No college: 785 (66.3)<br>Some college: 399 (33.7) | NR | NR | Medicaid: 1030 (87) | NR | V: 698 (59.0)<br>C: 486 (41.0) | Preterm birth: 234 (19.8) |
| Austin, 2022, 34974107 | 18-24: (44.8)<br>25-29: (28.3)<br>30-34: (16.9)<br>>35: (9.9) | NR | W: (43.2)<br>B: (20.5)<br>H: (28.9)<br>O1: (7.5) | Less than HS (26.5)<br>HS diploma or GED (38.9)<br>Some college (28.1)<br>College degree or more (6.5) | NR | NR | Medicaid: (74.2) | NR | NR | NR |
| Brant, 2021, 34619694 | 30.3 (5.5) | 32.6 (6.8) | W: 5526 (64.5)<br>B:1592 (18.7)<br>H: 459 (5.4)<br>O: 939 (11.4) | NR | NR | NR | Medicaid: 2840 (33.3) | HTN: 768 (9) | V: 5902 (69.3)<br>C: 2614 (30.7) | Stillbirth: 0<br>Spontaneous or induced abortion: 0<br>Preterm birth: 695 (8.2)<br>Neonatal death: 0 |
| Caudillo, 2022, 35488950 | <20: (5.9)<br>20-24: (20.7)<br>25-29: (30.5)<br>30-34: (28.3)<br>≥35: (14.4) | NR | W: (61.9)<br>B: (13.2)<br>A: (4.6)<br>H: (16.3)<br>O1: (4.0) | <HS (13.3)<br>HS (23.6)<br>Some college (28.8)<br>Bachelors or more (34.3) | NR | NR | Medicaid: (42.8) | NR | V: (67.9)<br>C: (32.1) | NR |
| Cilenti, 2015, 25627330 | NR | NR | NR | NR | NR | NR | Medicaid: NC<br>Medicaid: 1969 (100) | NR | NR | NR |

© 2023 Saldanha IJ et al. *JAMA Network Open.*

| Author, Year, PMID | Age in Years, Mean (SD) or as Specified | BMI, Mean (SD) or as Specified | Race, N (%) | Educational Attainment in Years, Mean (SD) or as Specified | Employment Status | SES | Miscellaneous N (%) | Chronic Conditions | Delivery Type | Offspring Characteristics |
|---|---|---|---|---|---|---|---|---|---|---|
| DeSisto, 2020, 32335806 | <20: (11)<br>20-24: (31.7)<br>25-29: (29.6)<br>30-34: (18.5)<br>>35: (9.3) | NR | W: (55.6)<br>B: (17.4)<br>A: (13.4)<br>H: (13.7) | <High school/GED (19.8) High school diploma/GED (38.9)<br>>High school/GED (40.8)<br>Unknown (0.6) | NR | NR | Medicaid: (100) | HDP: Gestational HTN (5.3)<br>HTN: Pre-pregnancy HNT (2.3)<br>GD: (6.6)<br>DM: Pre-pregnancy diabetes (1.2) | V: (74.4)<br>C: (25.6) | Multiple births (1.2)<br>Stillbirth (0)<br>Spontaneous or induced abortion (0)<br>Preterm birth (9.3) |
| Dunlop, 2020, 32958368 | 20-24: 61270 (44.3)<br>25-34: 67255 (48.6)<br>35-44: 9901 (7.1) | NR | W: 87855 (63.5)<br>B: 41313 (29.8)<br>H: 6121 (4.4)<br>O: 3151 (2.3) | Unknown: 1252 (0.9)<br>College graduate: 6663 (4.8)<br>Some college: 54028 (39)<br>HS graduate: 52157 (37.5)<br>Less than HS graduate: 24326 (17.8) | NR | NR | Medicaid: Ohio Medicaid: NR (100) | NR | NR | NR |
| Eliason, 2021, 34870677 | 18-24: 1862 (37)<br>25-30: 1483 (29.5)<br>30-34: 1066 (21.2)<br>35-39: 479 (9.5)<br>≥40: 143 (2.8) | NR | W: 2413 (47.9)<br>B: 730 (14.5)<br>A: 144 (2.8)<br>H: 973 (19.3)<br>O1: 315 (6.3) | HS or less 2472 (49.1)<br>More than HS 2494 (49.5)<br>Missing 68 (1.4) | NR | NR | NR | NR | NR | NR |
| Eliason, 2022, 35259409 | 18-24: (49.6)<br>25-29: (28.9)<br>30-34: (14.3)<br>35-39: (5.6)<br>≥40: (1.6) | NR | W: (65.5)<br>B: (14.7)<br>A: (1.5)<br>H: (12.8)<br>O1: (4.6) | HS or less (56.1)<br>More than HS (42.8)<br>NR (1.1) | NR | NR | NR | NR | NR | NR |
| Gordon, 2020, 31905073 | 19-24: 27395 (41.1)<br>25-39: 38125 (57.2)<br>40-53: 1152 (1.7) | NR | NR | NR | NR | NR | Medicaid: 66672 (100) | NR | NR | Stillbirth: 0<br>Spontaneous or induced abortion: 0<br>Neonatal death: 0 |

© 2023 Saldanha IJ et al. *JAMA Network Open.*

| Author, Year, PMID | Age in Years, Mean (SD) or as Specified | BMI, Mean (SD) or as Specified | Race, N (%) | Educational Attainment in Years, Mean (SD) or as Specified | Employment Status | SES | Miscellaneous N (%) | Chronic Conditions | Delivery Type | Offspring Characteristics |
|---|---|---|---|---|---|---|---|---|---|---|
| Koch, 2022, 35588793 | 27.6 (5.9) | NR | W: 2014 (32.3)<br>B: 3351 (53.8)<br>A: 233 (3.7)<br>H: 265 (4.3) | NR | NR | NR | Medicaid: 3902 (62.6) | NR | V: 3858 (61.9)<br>C: 2354 (37.8) | NR |
| Kozhimannil, 2011, 21485419 | 33.0 (95% CI 32.8, 33.2) | NR | NR | NR | Employed: 2509 (100) | Low*: 417 (16.6)<br>Not low*: 2092 (83.4) | Medicaid: 0 (0) | GD: 99 (4) | V: 1658 (66.1)<br>C: 851 (33.9) | Preterm birth: 233 (9.3) |
| Kramer, 2021, 33849768 | <20: 3803 (8.4)<br>20-24: 12876 (28.5)<br>25-29: 14639 (32.4)<br>30-34: 9201 (20.4)<br>≥35: 4681 (10.4) | NR | W: 22562 (49.9)<br>B: 10517 (23.3)<br>H: 7696 (17)<br>O: 4425 (9.8) | NR | NR | NR | Medicaid: 45200 (100) | NR | NR | NR |
| Liberty, 2020, 31846612 | 25.0 (5.4) | NR | W: 83788 (44.7)<br>B: 86869 (46.3)<br>H: 6780 (3.6)<br>O1: 1769 (0.9) | NR | NR | NR | Medicaid: 164004 (100) | HDP: 12354 (6.6)<br>HTN: Chronic HTN 5586 (3)<br>GD: 10072 (5.4)<br>DM: Pre-pregnancy diabetes 1958 (1) | V: 124451 (66.4)<br>C: 63064 (33.6) | Multiple births: (0)<br>Preterm birth 20447 (10.9) |
| Margerison, 2021, 34606358 | NR | NR | W: 27970 (49.1)<br>B: 9969 (17.5)<br>A: 1196 (2.1)<br>H: 13890 (24.4)<br>O1: 1709 (3)<br>O2: 2507 (4.4) | NR | NR | NR | Medicaid: 31957 (56.1) | NR | NR | NR |

© 2023 Saldanha IJ et al. *JAMA Network Open.*

| Author, Year, PMID | Age in Years, Mean (SD) or as Specified | BMI, Mean (SD) or as Specified | Race, N (%) | Educational Attainment in Years, Mean (SD) or as Specified | Employment Status | SES | Miscellaneous N (%) | Chronic Conditions | Delivery Type | Offspring Characteristics |
|---|---|---|---|---|---|---|---|---|---|---|
| Myerson, 2020, 33136489 | 20-24: 5680 (37.7)<br>25-29: 4888 (32.5)<br>30-34: 2900 (19.3)<br>35-39: 1226 (8.1)<br>40+: 326 (2.2) | NR | W: 7524 (50)<br>B: 2608 (17.3)<br>A: 1469 (9.8)<br>H: 3459 (23) | <12 years: 3392 (22.5)<br>12 years: 5605 (37.2)<br>13-15 years: 4799 (31.9)<br>≥16 years: 1480 (9.8) | NR | NR | NR | NR | NR | Stillbirth: 0<br>Neonatal death: 0<br>Congenital anomalies: 0 |
| Okoroh, 2018, 29530670 | NR | NR | NR | NR | NR | NR | Medicaid: 57894 (100) | NR | NR | Stillbirth: 0<br>Spontaneous or induced abortion: 0<br>Neonatal death: 0 |
| Pace, 2022, 34908011 | NR | NR | NR | NR | NR | NR | Medicaid: (100) | NR | NR | NR |
| Redd, 2019, 30484739 | ≤20: 6365 (9)<br>20-24: 16390 (22)<br>25-34: 39223 (52)<br>≥35: 13097 (17) | NR | W: 44084 (59)<br>B: 12631 (17)<br>A: 5357 (7)<br>O1: 12329 (17)<br>O2: NR | Some HS: 12274 (16)<br>HS graduate: 18465 (25)<br>Some college: 20787 (28)<br>College graduate: 23108 (31) | NR | NR | Medicaid: 13862 (18)<br>WIC during pregnancy: 35942 (48) | NR | NR | NR |
| Rodriguez, 2008, 18692614 | 26 (NR) | NR | NR | NR | NR | NR | Immigrants on Emergency Medicaid: 6286 (54.5) | NR | V: 8520 (73.9)<br>C: 3006 (26.1) | NR |
| Rodriguez, 2021, 34910148 | 29.4 (6)<br><br><20: 1172 (4.2)<br>20-34: 20449 (73.9)<br>≥35: 6046 (21.9) | NR | W: 1005 (3.6)<br>B: 473 (1.7)<br>A: 1367 (4.9)<br>H: 18408 (66.5)<br>O1: 5759 (20.8)<br>O2: 70 (0.3)<br>O3: 585 (2.1) | NR | NR | NR | Immigrant: non-citizens 27667 (100)<br>Medicaid: Emergency Medicaid Coverage 27667 (100) | NR | V: 20043 (72.4)<br>C: 7624 (27.6) | Preterm birth 2165 (7.8) |

© 2023 Saldanha IJ et al. *JAMA Network Open.*

| Author, Year, PMID | Age in Years, Mean (SD) or as Specified | BMI, Mean (SD) or as Specified | Race, N (%) | Educational Attainment in Years, Mean (SD) or as Specified | Employment Status | SES | Miscellaneous N (%) | Chronic Conditions | Delivery Type | Offspring Characteristics |
|---|---|---|---|---|---|---|---|---|---|---|
| Schuster, 2022, 34670222 | <25: 2341 (24.5)<br>25-34: 6 (65.2)<br>≥35: 1086 (10.3) | NR | W: 20382 (82.7)<br>B: 536 (5.2)<br>O: 1741 (12) | <HS: 614 (5.1)<br>HS: 1997 (18.8)<br>Some college: 3691 (41.1)<br>At least Bachelor's degree: 3060 (35) | NR | Household income 100-250% of federal poverty: 6984 (73)<br>Household income 251-400% of federal poverty: 2488 (27) | Medicaid: 2082 (20.6) | HTN: 449 (3.6)<br>DM: 243 (2) | NR | NICU: 2207 (11.8) |
| Smith, 2021, 34109490 | NR | NR | NR | NR | NR | NR | Medicaid: 4417 (78.2) | NR | NR | NR |
| Steenland, 2021a, 33523747 | 24.9 (5.5)<br>12-19: 23778 (15.4)<br>20-50: 13 (84.6) | NR | W: 65770 (42.8)<br>B: 65958 (42.9)<br>H: 15584 (10.1)<br>O: 6335 (4.1) | NR | NR | NR | Medicaid: 154163 (100) | NR | NR | NR |
| Steenland, 2021b, 35977301 | 27.1<br><br>19-24: (44.5)<br>25-30: (35.5)<br>31-35: (12.3)<br>36-50: (8.7) | NR | W: (62.3)<br>B: (24.3)<br>H: (8.6)<br>O1: (4.8) | Less than college (88.2)<br>College or higher (11.8) | NR | NR | NR | NR | V: (66.7)<br>C: (33.3) | NR |
| Symum, 2022, 35628011 | Median (28)<br><br><18: 27026 (1.8)<br>18-30: 858875 (57.2)<br>30-40: 566371 (37.8)<br>>40: 47722 (3.2) | NR | W: 724174 (48.3)<br>B: 347720 (23.2)<br>H: 329438 (22.9)<br>O1: 79979 (5.3) | NR | NR | NR | Medicaid beneficiaries (54.6) | NR | V: 915390 (61.1)<br>C: 584604 (38.9) | Preterm birth 122566 (8.1) |

© 2023 Saldanha IJ et al. *JAMA Network Open.*

| Author, Year, PMID | Age in Years, Mean (SD) or as Specified | BMI, Mean (SD) or as Specified | Race, N (%) | Educational Attainment in Years, Mean (SD) or as Specified | Employment Status | SES | Miscellaneous N (%) | Chronic Conditions | Delivery Type | Offspring Characteristics |
|---|---|---|---|---|---|---|---|---|---|---|
| Taylor, 2020, 31397625 | 18-24: 2088 (21.7)<br>25-34: 5698 (59.3)<br>≥35: 1827 (19) | Under-weight: 94 (1.0)<br>Normal: 3586 (37.3)<br>Overweight: 2897 (30.1)<br>With obesity: 2400 (25.0)<br>With severe obesity: 636 (6.6) | W: 2709 (28.2)<br>B: 2593 (27.0)<br>H: 2881 (30.0)<br>O: 1430 (14.9) | NR | NR | NR | Medicaid: 4990 (51.9) | HTN: 269 (2.8)<br>DM: 120 (1.2) | NR | Stillbirth: 0<br>Spontaneous or induced abortion: 0<br>Neonatal death: 0 |
| Wang, 2022, 35592081 | 26.9 (5.5)<br><br>14-24: 3107 (35)<br>25-34: 4704 (53)<br>35-48: 1065 (12) | NR | NR | NR | NR | NR | NR | HTN: 440 (5)<br>DM: 204 (2.3)<br>Asthma 661 (7.4) | NR | Multiple births: (0)<br>Preterm birth 1934 (21.8) |

Abbreviations: A = Asian, B = Black, BMI = body mass index, C = Cesarean delivery, CV = cardiovascular, DM = diabetes mellitus, GD = gestational diabetes, H = Hispanic, HS = high school, HTN = hypertension, O = Other, PMID = PubMed ID, SD = standard deviation, SES = socioeconomic status, V = vaginal delivery, W = White, WIC= women, Infants, and Children

*Defined as living in a census tract with either >25% of adults having less than a HS education or >10% of households living below the poverty level.

No studies reported on sexual/gender identities or substance use disorders.

© 2023 Saldanha IJ et al. *JAMA Network Open.*

**eAppendix 4. Risk of Bias Assessment—Confounding and Selection Bias**

| Author, Year, PMID | 1.1 Potential for Any Confounding? | 1.2 Potential for Time-Varying Confounding? | 1.3 Intervention Switches Related to Prognostic Factors? | 1.4 Appropriate Analysis Method for [illegible] | 1.5 Appropriate Confounding Variables Used? | 1.6 Inappropriate Control of Post-Intervention Variables? | Judgment – Risk of Bias Related to Confounding | 2.1 Participant Selection Based on Post-Intervention Variables? | 2.2 Post-Intervention Variables Associated with Intervention? | 2.3 Post-Intervention Variables Associated with Outcome? | 2.4 Start and Follow-Up (Duration) Coincide | 2.5 Appropriate Adjustment for Selection Bias | Judgment – Risk of Bias Related to Selection Bias |
|---|---|---|---|---|---|---|---|---|---|---|---|---|---|
| Arora, 2018, 29490290 | Y | N | N/A | N | N/A | N | **Serious** | PN | N/A | N/A | Y | N/A | **Low** |
| Austin, 2022, 34974107 | Y | N | N/A | Y | Y | N | **Low** | PN | N/A | N/A | PY | N/A | **Low** |
| Brant, 2021, 34619694 | Y | N | N/A | Y | PY | N | **Low** | N | N/A | N/A | Y | N/A | **Low** |
| Caudillo, 2022, 35488950 | Y | N | N/A | PY | PY | PN | **Low** | N | N/A | N/A | PY | N/A | **Low** |
| Cilenti, 2015, 25627330 | Y | N | N/A | Y | PY | PN | **Low** | N | N/A | N/A | Y | N/A | **Low** |
| DeSisto, 2020, 32335806 | Y | N | N/A | PY | PY | PN | **Low** | PN | N/A | N/A | Y | N/A | **Low** |
| Dunlop, 2020, 32958368 | Y | N | N/A | Y | Y | PN | **Low** | PN | N/A | N/A | Y | N/A | **Low** |
| Eliason, 2021, 34870677 | Y | N | N/A | Y | Y | N | **Low** | PN | N/A | N/A | Y | N/A | **Low** |
| Eliason, 2022, 35259409 | Y | N | N/A | Y | Y | PN | **Low** | N | N/A | N/A | Y | N/A | **Low** |
| Gordon, 2020, 31905073 | Y | N | N/A | Y | Y | N | **Low** | N | N/A | N/A | Y | N/A | **Low** |
| Koch, 2022, 35588793 | Y | N | N/A | PY | Y | N | **Low** | PN | N/A | N/A | Y | N/A | **Low** |
| Kozhimannil, 2011, 21485419 | Y | N | N/A | Y | Y | N | **Low** | PN | N/A | N/A | Y | PY | **Low** |
| Kramer, 2021, 33849768 | Y | N | N/A | Y | Y | N | **Low** | PN | N/A | N/A | Y | N/A | **Low** |
| Liberty, 2020, 31846612 | Y | N | N/A | Y | PY | PN | **Low** | PN | N/A | N/A | PY | N/A | **Low** |
| Margerison, 2021, 34606358 | Y | N | N/A | Y | Y | N | **Low** | PN | N/A | N/A | Y | N/A | **Low** |
| Myerson, 2020, 33136489 | Y | N | N/A | Y | PY | N | **Low** | PN | N/A | N/A | Y | N/A | **Low** |
| Okoroh, 2018, 29530670 | Y | N | N/A | PN | N/A | PN | **Moderate** | PN | N/A | N/A | Y | N/A | **Low** |
| Pace, 2022, 34908011 | Y | N | N/A | PN | N/A | PN | **Moderate** | PN | N/A | N/A | PY | N/A | **Low** |
| Redd, 2019, 30484739 | Y | N | N/A | Y | Y | PN | **Low** | PN | N/A | N/A | Y | N/A | **Low** |
| Rodriguez, 2008, 18692614 | Y | N | N/A | PN | N/A | PN | **Moderate** | PN | N/A | N/A | Y | N/A | **Low** |
| Rodriguez, 2021, 34910148 | Y | N | N/A | PY | Y | N | **Low** | N | N/A | N/A | PY | N/A | **Low** |
| Schuster, 2022, 34670222 | Y | N | N/A | N | N/A | PN | **Serious** | N | N/A | N/A | Y | N/A | **Low** |
| Smith, 2021, 34109490 | Y | N | N/A | N | N/A | PN | **Serious** | PN | N/A | N/A | Y | N/A | **Low** |
| Steenland, 2021a, 33523747 | Y | N | N/A | PN | N/A | N | **Moderate** | PN | N/A | N/A | Y | N/A | **Low** |
| Steenland, 2021b, 35977301 | Y | N | N/A | PN | N/A | N | **Moderate** | PN | N/A | N/A | Y | N/A | **Low** |
| Symum, 2022, 35628011 | Y | N | N/A | PY | Y | N | **Low** | N | N/A | N/A | Y | N/A | **Low** |
| Taylor, 2020, 31397625 | Y | N | N/A | Y | PY | N | **Low** | PN | N/A | N/A | Y | N/A | **Low** |
| Wang, 2022, 35592081 | Y | N | N/A | N | N/A | PN | **Serious** | PN | N/A | N/A | PY | N/A | **Low** |

© 2023 Saldanha IJ et al. *JAMA Network Open.*

Abbreviations: N/A = Not applicable, NI = no information, PMID = PubMed identifier, PN = probably no, PY = probably yes, Y = yes.

Judgements are color coded for emphasis only. The colors do not impart unique information. Signaling questions are not color coded for simplicity and because they are only used to inform the judgements.

Responses to Risk of Bias in Nonrandomized Studies of Interventions (ROBINS-I) signaling questions 1.1 to 1.6 and 2.1 to 2.5 are in regular font. Each item is rated as Yes, PY, NI, PN, No, or N/A.

Overall judgements about confounding and selection bias are in **bold font**. Each judgement is rated as **Low**, **Moderate**, **Serious**, **Critical**, or **NI**.

© 2023 Saldanha IJ et al. *JAMA Network Open.*

**eAppendix 5. Risk of Bias Assessment—Assessment of Remaining Biases, Quality, and Overall Risk of Bias**

| Study, Year, PMID | Blinding of Participants/ Care Providers | Blinding of Outcome Assessors | Incomplete Outcome Data | Selective Outcome Reporting | Other Bias | Overall Risk of Bias |
|---|---|---|---|---|---|---|
| Arora, 2018, 29490290 | High | High | Low | Low | Low | **HIGH** |
| Austin, 2022, 34974107 | High | High | Low | Low | Low | **MODERATE** |
| Brant, 2021, 34619694 | High | High | Low | Low | Low | **MODERATE** |
| Caudillo, 2022, 35488950 | High | High | Low | Low | Low | **MODERATE** |
| Cilenti, 2015, 25627330 | High | High | Low | Low | Low | **MODERATE** |
| DeSisto, 2020, 32335806 | High | High | Low | Low | Low | **MODERATE** |
| Dunlop, 2020, 32958368 | High | High | Low | Low | Low | **MODERATE** |
| Eliason, 2021, 34870677 | High | High | Low | Low | Low | **MODERATE** |
| Eliason, 2022, 35259409 | High | High | Low | Low | Low | **MODERATE** |
| Gordon, 2020, 31905073 | High | High | Low | Low | Low | **MODERATE** |
| Koch, 2022, 35588793 | High | High | Low | Low | Low | **MODERATE** |
| Kozhimannil, 2011, 21485419 | High | High | Low | Low | Low | **MODERATE** |
| Kramer, 2021, 33849768 | High | High | Low | Low | Low | **MODERATE** |
| Liberty, 2020, 31846612 | High | High | Low | Low | Low | **MODERATE** |
| Margerison, 2021, 34606358 | High | High | Low | Low | Low | **MODERATE** |
| Myerson, 2020, 33136489 | High | High | Low | Low | Low | **MODERATE** |
| Okoroh, 2018, 29530670 | High | High | Low | Low | Low | **HIGH** |
| Pace, 2022, 34908011 | High | High | Low | Low | Low | **HIGH** |
| Redd, 2019, 30484739 | High | High | Low | Low | Low | **MODERATE** |
| Rodriguez, 2008, 18692614 | High | High | Low | Low | Low | **HIGH** |
| Rodriguez, 2021, 34910148 | High | High | Low | Low | Low | **MODERATE** |
| Schuster, 2022, 34670222 | High | High | Low | Low | Low | **HIGH** |
| Smith, 2021, 34109490 | High | High | Low | Low | Low | **HIGH** |
| Steenland, 2021a, 33523747 | High | High | Low | Low | Low | **HIGH** |
| Steenland, 2021b, 35977301 | High | High | Low | Low | Low | **HIGH** |
| Symum, 2022, 35628011 | High | High | Low | Low | Low | **MODERATE** |
| Taylor, 2020, 31397625 | High | High | Low | Low | Low | **MODERATE** |
| Wang, 2022, 35592081 | High | High | Low | Low | Low | **HIGH** |

Abbreviations: PMID = PubMed identifier.

Judgements are color coded for emphasis only. The colors do not impart unique information.

Overall judgements are in **bold font**. Each study is rated as **LOW**, **MODERATE**, **HIGH**, or **NI**. Overall risk of bias is low if either participants or outcome assessors are blinded and all other domains are at low risk of bias (no instances in this table), moderate if neither participants nor outcome assessors are blinded and all other domains are at low risk of bias, and high risk of bias otherwise.

© 2023 Saldanha IJ et al. *JAMA Network Open.*

**eAppendix 6. Continuous Outcomes, Healthcare Utilization**

| Study, Year, PMID | Overall RoB | Outcome Description | Arm Name | Sub-group | Mean at Baseline (SD or 95% CI) | Followup Time-Point | Mean at Followup (SD or 95% CI) | MD within arms (95% CI) | Effect size (95% CI) | Reported P Value |
|---|---|---|---|---|---|---|---|---|---|---|
| Cilenti, 2015, 25627330 | Moderate | Number of PP visits | More comprehensive insurance | All | NR | 3 mo | 4.3 (NR) | N/A | adjMD 1.6 (NR) | <0.001 |
| | . | . | Less comprehensive insurance | . | NR | . | 2.7 (NR) | N/A | Ref | Ref |
| Gordon, 2020, 31905073 | Moderate | Number of outpatient visits by 1 mo | More comprehensive insurance | All | 1.40 (NR) | After CO's expansion | 1.38 (NR) | −0.02 (NR) | adjNMD 0 (NR) | NS |
| | . | . | Less comprehensive insurance | . | 0.73 (NR) | . | 0.7 (NR) | −0.20 (NR) | Ref | Ref |
| | . | Number of outpatient visits by 3 mo | More comprehensive insurance | All | 0.44 (NR) | After CO's expansion | 0.49 (NR) | 0.05 (NR) | adjNMD 0.10 (NR) | <0.0001 |
| | . | . | Less comprehensive insurance | . | 0.32 (NR) | . | 0.27 (NR) | −0.05 (NR) | Ref | Ref |
| | . | Number of outpatient visits by 6 mo | More comprehensive insurance | All | 3 (NR) | After CO's expansion | 3.3 (NR) | 0.30 (NR) | adjNMD 0.52 (NR) | <0.01 |
| | . | . | Less comprehensive insurance | . | 2 (NR) | . | 1.8 (NR) | −0.20 (NR) | Ref | Ref |
| | . | . | More comprehensive insurance | Severe PP morbidity | 2.7 (NR) | After CO's expansion | 3.4 (NR) | 0.70 (NR) | adjNMD 1.25 (NR) | <0.01 |
| | . | . | Less comprehensive insurance | . | 1.8 (NR) | . | 1.6 (NR) | −0.20 (NR) | Ref | Ref |
| Steenland, 2021b, 35977301 | High | Number of outpatient visits by 2 mo | More comprehensive insurance | All | NR | 2 mo | NR | NR | adjMD 0.2 (0.1, 0.3) | <0.001 |
| | . | . | Less comprehensive insurance | . | NR | . | NR | NR | Ref | Ref |
| | . | Number of outpatient visits by 6 mo | More comprehensive insurance | All | NR | 6 mo | NR | | adjMD 0.9 (0.7, 1.1) | <0.001 |
| | . | . | Less comprehensive insurance | . | NR | . | . | NR | Ref | Ref |

Abbreviations: adj = adjusted, CI = confidence interval, mo = months, MD = mean difference, NMD = net mean difference, NR = not reported, PMID = PubMed identifier, PP = postpartum, Ref = reference arm, RoB = risk of bias, SD = standard deviation

© 2023 Saldanha IJ et al. *JAMA Network Open.*

**eAppendix 7. Categorical Outcomes, Healthcare Utilization**

| Study, Publication Year, PMID | Overall RoB | Outcome Description | Arm Name | Sub-group | Time- point | n/N (%) | Effect Size (95% CI) | Reported P value |
|---|---|---|---|---|---|---|---|---|
| DeSisto, 2020, 32335806 | Moderate | PP visit, cervical cytology, IUD insertion, or a bundled service | More comprehensive insurance | All | 8 wk | NR | adjRD 6.27 (5.72, 6.82) | NR |
| | . | . | Less comprehensive insurance | . | . | NR | Ref | Ref |
| | . | PP visit, cervical cytology, or IUD insertion | More comprehensive insurance | All | 8 wk | NR | adjRD 12.0 (11.2, 12.7) | NR |
| | . | . | Less comprehensive insurance | . | . | NR | Ref | Ref |
| Dunlop, 2020, 32958368 | Moderate | PP visit attendance | More comprehensive insurance | Income-eligible | 6 mo | 1580/36603 (37.1) | OR, adjusted marginal effect 5.09 | <0.01 |
| | . | . | Less comprehensive insurance | . | . | 1458/46428 (31.5) | Ref | Ref |
| | . | . | More comprehensive insurance | Pregnancy-eligible | 6 mo | 5430/17784 (30.5) | OR, adjusted marginal effect −0.16 | NS |
| | . | . | Less comprehensive insurance | . | . | 12082/37521 (32.2) | Ref | Ref |
| Eliason, 2021, 34870677 | Moderate | PP visit attendance | More comprehensive insurance | All | Before policy change | 2460/3389 (89.6) | NR | NR |
| | . | . | Less comprehensive insurance | . | . | 1307/1645 (90.5) | NR | NR |
| | . | . | More comprehensive insurance | . | After policy change | 3630/4197 (87.6) | adjNPD 0.3 (-3.1, 3.9) | NR |
| | . | . | Less comprehensive insurance | . | . | 1903/2201 (86.9) | Ref | Ref |
| Kozhimannil, 2011, 21485419 | Moderate | PP visit attendance 21-56 days | More comprehensive insurance | All | Before policy change | 418/711 (58.8) | NR | NR |
| | . | . | Less comprehensive insurance | . | . | 52/86 (60.5) | NR | NR |
| | . | . | More comprehensive insurance | . | After policy change | 893/1569 (56.9) | Ref | Ref |
| | . | . | Less comprehensive insurance | . | . | 73/143 (51.1) | adjOR 0.74 (0.42, 1.32) | NR |
| Liberty, 2020, 31846612 | Moderate | PP visit attendance | More comprehensive insurance | All | 6 wk | 83621/129645 (64.5) | NR | NR |
| | . | . | Less comprehensive insurance | . | . | 38652/57862 (66.8) | NR | NR |
| Rodriguez, 2021, 34910148 | Moderate | PP visit within 2mo | More comprehensive insurance | All | Before policy change | 1050/11988 (8.8) | NR | NR |
| | . | . | Less comprehensive insurance | . | . | NR | NR | NR |

© 2023 Saldanha IJ et al. *JAMA Network Open.*

| Study, Publication Year, PMID | Overall RoB | Outcome Description | Arm Name | Sub-group | Time- point | n/N (%) | Effect Size (95% CI) | Reported P value |
|---|---|---|---|---|---|---|---|---|
| | . | . | More comprehensive insurance | . | After policy change | 1933/3477 (55.6) | AdjNPD 47.9 (41.3, 54.6) | NR |
| | . | . | Less comprehensive insurance | . | . | NR | Ref | NR |
| Symum, 2022, 35628011 | Moderate | Preventable readmissions | More comprehensive insurance | All | 1.5 mo | NR | IRR 0.86 (0.80, 0.93) | NR |
| | . | . | Less comprehensive insurance | . | . | NR | Ref | NR |
| | . | ED visits | More comprehensive insurance | All | 1.5 mo | NR | IRR 0.87 (0.82, 0.93) | NR |
| | . | . | Less comprehensive insurance | . | . | NR | Ref | NR |
| Taylor, 2020, 31397625 | Moderate | PP visit attendance | Commercial insurance | All | 6 wk | 2715/3998 (67.9) | Ref | Ref |
| | . | . | Medicaid insurance | . | . | 2455/4990 (49.2) | adjOR 0.65 (0.58, 0.74) | <0.01 |
| | . | . | No insurance | . | . | 75/182 (41.2) | adjOR 0.42 (0.34, 0.51) | <0.01 |
| Wang, 2022, 35592081 | High | Attendance at PP visits | More comprehensive insurance | All | 3 mo | (66.4) | NR | NR |
| | . | . | Less comprehensive insurance | . | . | (69.4) | NR | NR |
| | . | . | More comprehensive insurance | All | 3-6 mo | (6.7) | NR | NR |
| | . | . | Less comprehensive insurance | . | . | (3.2) | NR | NR |

Abbreviations: adj = adjusted, CI = confidence interval, ED = emergency department, IRR = incidence rate ratio, IUD = intrauterine device, NR = not reported, NPD = net prevalence difference, OR = odds ratio, PMID = PubMed identifier, PP = postpartum, Ref = reference arm, RoB = risk of bias, wk = weeks

© 2023 Saldanha IJ et al. *JAMA Network Open.*

**eAppendix 8. Categorical Outcomes, Mental Health Symptoms**

| Study, Year, PMID | Overall ROB | Outcome Description | Arm Name | Timepoint | n/N (%) | Effect Size (95% CI) | Reported P value |
|---|---|---|---|---|---|---|---|
| Austin, 2022, 34974107 | Moderate | "Always" or "often" experiencing depressive symptoms | More comprehensive insurance | Before Medicaid expansion | 25074/30250 (86.9) | NR | NR |
| | . | . | Less comprehensive insurance | . | 14222/17736 (83.5) | NR | NR |
| | . | . | More comprehensive insurance | After Medicaid expansion | 18635/20935 (91.4) | adjPR 0.93 (0.80, 1.07) | NR |
| | . | . | Less comprehensive insurance | . | 12000/13792 (87.8) | Ref | Ref |
| Margerison, 2021, 34606358 | Moderate | "Always" or "often" felt down/ depressed/hopeless or had little interest/pleasure in doing things | More comprehensive insurance | Before Medicaid expansion | NR/NR (15.2) | NR | NR |
| | . | . | Less comprehensive insurance | . | NR/NR (17.6) | NR | NR |
| | . | . | More comprehensive insurance | After Medicaid expansion | NR/NR (15.5) | adjNPD 0.0 | NS |
| | . | . | Less comprehensive insurance | . | NR/NR (18.6) | Ref | Ref |
| Schuster, 2022, 34670222 | High | "Always" or "often" felt down/ depressed/hopeless or had little interest/pleasure in doing things | More comprehensive insurance | PP | NR | adjPD −3.5% | 0.042 |
| | . | . | Less comprehensive insurance | . | NR | Ref | Ref |

Abbreviations: adj = adjusted, CI = confidence interval, NPD = net prevalence difference, NR = not reported, NS = not statistically significant, PD = prevalence difference, PMID = PubMed identifier, PP = postpartum, PR = prevalence ratio, Ref = reference arm, RD = risk difference, RoB = risk of bias
