# Supplementary material for: Health Insurance Coverage and Postpartum Outcomes in the US: A Systematic Review
Source: JAMA Netw Open. 2023 Jun 2;6(6):e2316536. doi: 10.1001/jamanetworkopen.2023.16536 (PMC10238947; doi:10.1001/jamanetworkopen.2023.16536)
Supplement: Supplement 2. — Data Sharing Statement [file jamanetwopen-e2316536-s002.pdf]

Data Sharing Statement

Saldanha. Health Insurance Coverage and Postpartum Outcomes in the US. *JAMA Netw Open*. Published June 02, 2023. doi:10.1001/jamanetworkopen.2023.16536

Data
Data available: Yes
Data types: Data (not involving human participants)
How to access data: Systematic Review Data Repository Plus (https://srdrplus.ahrq.gov)
When available: With publication

Supporting Documents
Document types: None

Additional Information
Who can access the data: Anyone requesting the data
Types of analyses: All extracted data
Mechanisms of data availability: Without investigator support
Any additional restrictions: Creative Commons License
